# MMGS: a novel genomic prediction framework to integrate genotype, environment and their interactions for multi-environment breeding trials

**DOI:** 10.1093/hr/uhag035

**Published:** 2026-03-16

**Authors:** Mingjia Zhu, Zeyu Zheng, Wei Liu, Yu Han, Wenjie Mou, Tongming Yin, Xiaogang Dai, Huaitong Wu, Yongzhi Yang, Yanjun Zan, Jianquan Liu

**Affiliations:** State Key Laboratory of Herbage Innovation and Grassland Agro-Ecosystem, College of Ecology, Lanzhou University, Lanzhou, China; State Key Laboratory of Herbage Innovation and Grassland Agro-Ecosystem, College of Ecology, Lanzhou University, Lanzhou, China; Yazhouwan National Laboratory (YNL), Sanya, Hainan, China; College of Life Sciences, China Sichuan University, Chengdu, Sichuan, China; College of Life Sciences, China Sichuan University, Chengdu, Sichuan, China; Departamento de Ciencias Agrarias y del Medio Natural, Escuela Politecnica Superior de Huesca, Universidad de Zaragoza, Huesca 22071, Spain; College of Forestry, Nanjing Forestry University, Nanjing, Jiangsu, China; College of Forestry, Nanjing Forestry University, Nanjing, Jiangsu, China; College of Forestry, Nanjing Forestry University, Nanjing, Jiangsu, China; State Key Laboratory of Herbage Innovation and Grassland Agro-Ecosystem, College of Ecology, Lanzhou University, Lanzhou, China; Integrated Science Lab, Department of Plant Physiology, Umeå Plant Science Center, Umeå University, Umeå 90736, Sweden; Tobacco Research Institute, Chinese Academy of Agricultural Sciences, Qingdao, China; State Key Laboratory of Herbage Innovation and Grassland Agro-Ecosystem, College of Ecology, Lanzhou University, Lanzhou, China

## Abstract

Accurately predicting the performance of trees and crops across diverse and changing climates is essential for matching genotypes to both current and future environments. Yet modelling the complex interplay among genotype, environment, and phenotype in multi-environment trials remains a major challenge. Here, we introduce a unified framework, polygenic environmental interaction (PEI), directly models genotype-by-environment interactions through integrating genotypes and environmental covariates. We implemented an ensemble of 15 estimators spanning parametric, non-parametric, and machine-learning approaches. We then benchmarked our framework against the classical reaction norm (RN) using three genetically distinct populations and three traits with variable genetic architectures. Furthermore, we released an open-source R package, Multiple-environments genomic selection (MMGS), on GitHub. Together, our study offers a flexible and computationally efficient approach for multi-environment genomic prediction, enhancing breeding efficiency, providing deeper insights into modelling the genotype-environment-phenotype continuum.

## Introduction

Both artificially cultivated trees and herbaceous crops exhibited plastic responses to changes in their surrounding environments, with one genotype performing well in one environment yet underperforming in another [1–3]. This variability poses a significant challenge in breeding crops and trees to meet the growing global food demand amid climate change [4]. To address this, breeders routinely screen for elite plants by subjecting germplasm and breeding materials to multiple-environment trials (METs) [5, 6]. Despite the advancements achieved through METs, several challenges persist, including the extensive number of genotypes tested in preliminary trials, the high associated costs and limited seed availability [7].

Over the past decades, genomic prediction has been increasingly applied to assess the overall performance and stability of genotypes across diverse environments, yet accurately capturing their overall stability remains challenging [8–10]. Through METs, it has been observed that variation in traits related to fitness and productivity is influenced by complex interactions between genotypes and the environment (G × E) [11–15]. In the absence of genetic mechanisms underlying variation in complex traits across environments, reaction norm (RN) assumes each genotype responds to changes in environment with a unique intercept and slope, representing distinct biological mechanisms [16–18]. Building upon this framework, an additional random effect was added to capture interactions between high-dimensional markers and environmental covariates (ECs) [19]. This extension integrated genetic relatedness into the estimation of regression slope and intercept, avoiding assuming each genotype is independent [20, 21]. Recently, an environment index was included in the RN framework, and Ridge regression best linear unbiased prediction (rr-BLUP) was used to predict the regression slope and intercept. With these advancements, environmental-specific predictions were achieved and prediction accuracy was significantly improved [20, 22]. However, further improvements are still needed to enhance the prediction of the cross-environment METs [23–25]. For example, modelling phenotypes in multiple environments through the RN framework assumes a linear relationship between genotypes and environments, which is not always supported by empirical data [21, 26]. In addition, recent studies have revealed a polygenic basis of phenotype plasticity, underpinned by interactions from core genes, polygenic background, and their interactions with environments. These advancements in how interaction between genotypes and environment co-determine the variation of complex traits have not yet been implemented for predicting complex traits across the environment. Moreover, previous frameworks only employed rr-BLUP for predicting parametric estimators within the RN framework. The vast number of parametric or non-parametric estimators, such as Bayesian, non-linear, and machine learning methods, have not been fully integrated into cross-environment predictions to account for variation in the underlying genetic basis of targeted traits [19, 22, 27].

To address these challenges, we implemented a polygenic environmental interaction (PEI) framework, which generalizes the factorial regression models by directly modelling G × E. Unlike existing frameworks, PEI explicitly integrates the Critical Environmental Regressor through Informed Search (CERIS) algorithm to identify the most informative ECs and incorporates a flexible ensemble of 15 parametric and non-parametric estimators (e.g. Bayesian, kernel, and machine learning estimators). This integration enables the framework to account for polygenic and potentially non-linear environmental responses while maintaining interpretability and computational tractability. To evaluate the performance of PEI, we compared our framework with the RN to evaluate the prediction accuracy and computational efficiency across three genetically and phenotypically diverse populations: an inbred wheat population [28], a Complete-diallel design plus Unbalanced Breeding-like Inter-Cross (CUBIC) maize population [29] and an F_1_ half-sib willow population. Furthermore, we released an open-source R package (multiple-environments genomic selection [MMGS]), available at GitHub (https://github.com/Ryougi-yukiro/MMGS), to facilitate its application in breeding programs. Overall, the PEI framework and MMGS package provide a unified, flexible, and computationally efficient platform that bridges classical G × E modelling and modern machine learning approaches, enhancing our capacity to predict genotype performance and stability across diverse and future environments.

## Results

### Two types of frameworks for cross-environment prediction in METs

**Figure 1:**
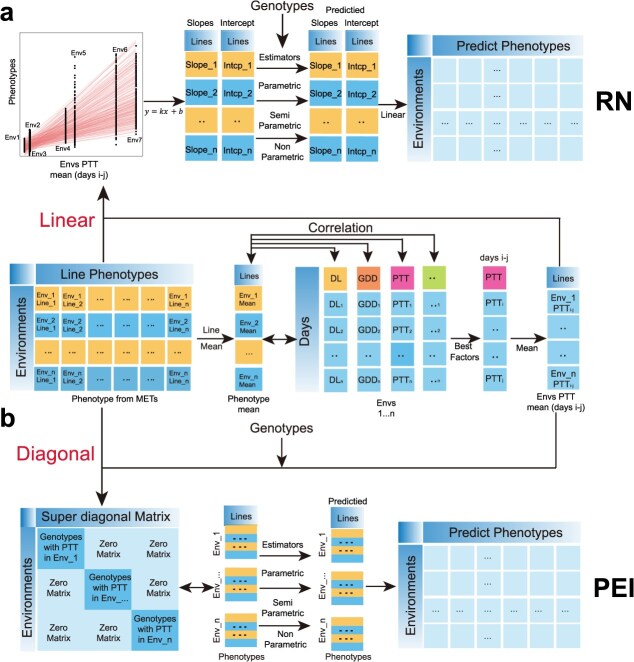
A schematic illustration of the two types of frameworks. (a) Concept and workflow of the Reaction Norm framework. (b) Concept and workflow of the PEI framework.

Previously, Jarquin *et al*. developed a framework for cross-environment genomic prediction by extending the RN framework with additional random effects to handle interactions between high-dimensional markers and ECs [18]. By accounting for genetic relatedness and environmental similarity, this framework significantly increased cross-environment genomic prediction accuracy. Building upon these progresses, a framework named CERIS-JRGA was developed [20, 22]. This framework includes four steps: (i) Applying the CERIS algorithm to identify an environmental index that captures the largest proportion of phenotypic variation. (ii) Regressing the observed phenotypes onto the identified environmental index to obtain an intercept and a slope estimate for each tested genotype. (iii) Treating intercept and slope as new ‘traits’ and performing genomic prediction via ridge regression to predict the intercept and slope for each untested genotype. (iv) Predicting phenotypes of the untested genotypes using the predicted intercept, slope and the environmental index (Fig. 1a).

Here, we implemented a second framework directly modelling the effects from genetic markers, environmental factors, and their interactions. This framework is based on recent insights into the polygenic basis of phenotype plasticity and directly modelling interaction between polygenic genetic background and environmental factors (PEI) [22, 30, 31]. Consistent with the RN framework, the PEI starts by identifying the key environmental index that best captures the phenotypic variation (Fig. 1b). We subsequently constructed a relationship matrix that includes not only block diagonal matrices assembled from genetic markers but also all pairwise interactions between these markers and the identified environmental index.

The RN framework employed a mixed linear model to transform multi-environmental phenotypic records into RN parameters, while the PEI framework directly models these measures by integrating genotype, environment index, and their interactions (Fig. 1). Consequently, the PEI framework fully leverages the interactions between genotypes and environmental factors, offering a more comprehensive approach to capturing genotype–environment dynamics.

Parametric estimators for both frameworks could be performed in the linear mixed model or using various machine learning approaches. We therefore implemented 15 estimators, with unique assumptions on underlying genetic architecture and variable computational efficacy. Overall, these estimators could be classified into three major categories: parametric, semi-parametric, and non-parametric [32]. The parametric estimators include mixed linear models, such as genomic best linear unbiased prediction (G-BLUP) [33], BayesA (BA), BayesB (BB) [34], BayesC (BC) [35], Bayesian ridge regression (BRR) [36], Bayesian LASSO (BL) [37], least absolute shrinkage and selection operator (LASSO) [38], ridge regression (RR) [39], rr-BLUP [34], and elastic net (EN) [40]. The semi-parametric estimators include the reproducing kernel Hilbert space (RKHS) and multiple kernel RKHS (MKRKHS) [41]. The non-parametric estimators include support vector machine (SVM) [42], random forest (RF) [43], and gradient boosting machine (GBM) [44, 45]. These frameworks and estimators were implemented in an R package named as MMGS, which has been publicly available at GitHub, along with a detailed tutorial summarized in Fig. S1.

Hereafter, we refer to the two prediction frameworks, RN and PEI, as frameworks, and various parameter estimators as estimators. Since prediction performance may vary depending on the population structure of interested population (i.e. inbred/outbred cross, generation, and number of founders), the genetic basis of targeted trait, the number of environments, parameter estimators used, etc., we selected multiple traits from a maize multi-parental advanced inter-cross population, a wheat inbred population and a willow F1 hybrid population to benchmark the prediction performance. For each population, three traits with varying genetic architecture were analysed with all 15 estimators. Prediction accuracy at each environment was calculated as average accuracy from 10-fold cross-validation with five replications. The averaged accuracy of all tested environments was used as an indicator of overall performance.

### Prediction accuracy for multiple traits in a maize multi-parental advanced inter-cross population multi-environment trial

**Figure 2:**
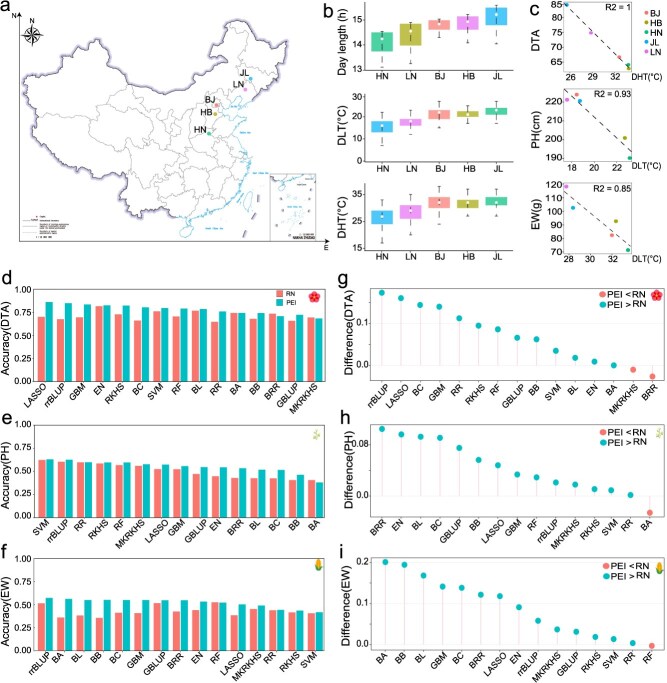
Prediction accuracy for multiple traits in a maize multiple parental advanced inter-cross multi-environment trial. (a) Geographical distribution of the five locations (Map Content Approval Number: GS (2019)1686); (b) Boxplot of the environmental factors at the five locations. (c) Performance of the correlation between the mean phenotypes and environmental index within optimal time windows across diverse locations. (d-f) Performance of the 15 estimators for the two frameworks on Days to Anthesis (DTA), Plant height (PH), and Ear weight (EW); (g-i) Comparison of the prediction accuracy between PEI and RN frameworks across 15 estimators on DTA, PH, and EW.

We first benchmarked the prediction performance using a maize multi-parental advanced inter-cross population (CUBIC) [29, 46]. This population was derived from 24 elite Chinese maize inbred lines from four divergent heterotic groups, and a total of 24 founders were crossed under a complete diallel cross mating design, omitting reciprocal crosses. A total of 1404 inbred maize lines were obtained and phenotyped at five locations in China's major maize production zone, covering longitudinal variation from E 114°01′ at Henan (HN) to E 125°18′ at Jilin (JL) and latitudinal variation from N 43°42′ at JL to N 35°27′ at HN (Fig. 2a) [47]. Furthermore, continuous monitoring of environmental data at the planting locations was conducted (Fig. 2b). Here, we assessed the prediction performance using three traits: days to anthesis (DTA), plant height (PH), and ear weight (EW) with distinct genetic architecture (Fig. S2, Table S1).

We applied the CERIS algorithm to search for an environmental index for each trait. Daily highest temperature (DHT) explained the largest amount of variation for DTA and PH, while daily lowest temperature (DLT) was correlated with EW with the highest correlation (Fig. 2c, Figs S3, S4  Table S2). These indexes were integrated into the two frameworks for predicting these traits across multiple environments. PEI framework exhibited superior prediction accuracy than that from the RN framework, with a 10%, 8.7%, and 20.6% increase in accuracy for DTA, PH, and EW (Fig. 2d–f, Table S2-S4). It is noteworthy that the optimal estimators varied between different frameworks. Specifically, for DTA, EN yielded the highest prediction accuracy for RN framework, whereas LASSO exhibited superior performance for PEI framework (Fig. 2d). In contrast, for PH, SVM outperformed other estimators for RN and PEI frameworks (Fig. 2e). In the case of EW, rr-BLUP demonstrated the highest accuracy for PEI framework, while RF achieved optimal performance for RN framework (Fig. 2f). Overall, most estimators for the PEI framework demonstrated improved prediction accuracy compared to those for the RN framework in this population (Fig. 2g–i).

### Prediction accuracy for multiple traits in an inbred wheat population multi-environment trial

**Figure 3:**
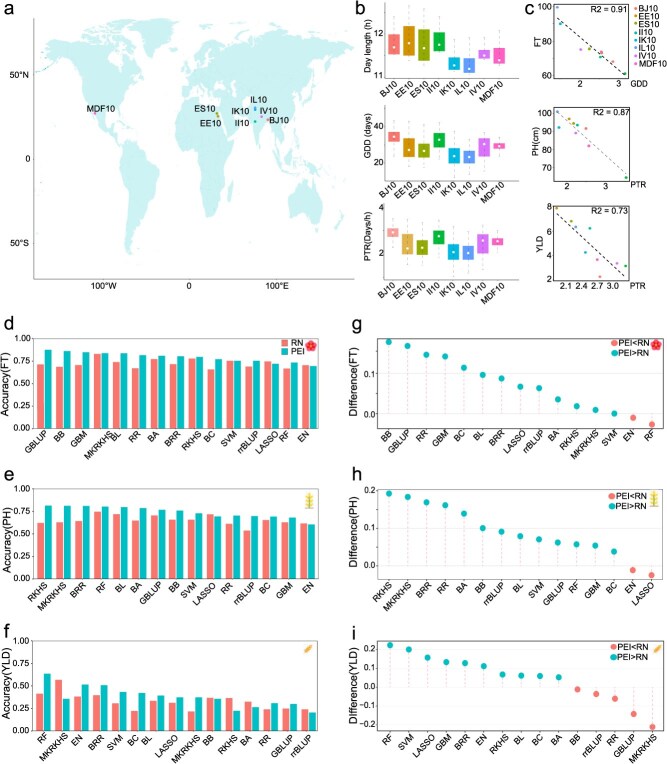
Prediction accuracy for multiple traits in an inbred wheat multi-environment trial. (a) Geographic distribution of experimental locations for the nine locations; (b) Boxplot of the environmental factors at the nine locations. (c) Performance of the correlation between the mean phenotypes and environmental index within optimal time windows across diverse locations. (d-f) Performance of the 15 estimators for the two frameworks on Flowering time (FT), Plant height (PH), and Yield (YLD); (g-i) Comparison of the prediction accuracy between PEI and RN frameworks across 15 estimators on FT, PH, and YLD.

We then benchmarked the prediction performance using an inbred wheat population from the International Maize and Wheat Improvement Center (CIMMYT). This population is part of the Wheat Association Mapping Initiative (WAMI) and was assembled from elite wheat nurseries distributed through the International Wheat Improvement Network (IWIN) [28, 48]. A total of 287 inbred wheat lines were genotyped and phenotyped at nine locations across major global production zones, including India in Asia, Mexico in North America, and Egypt in North Africa. These locations covered a wide range of geographical regions, from E 90°13′ at Joydebpur (BJ10) to W 109°33′ at Sonora (MDF10), and from N 15°15′ at Dharwad (ID10) to N 30°32′ at Ludhiana Fig. 3a(IL10) (Fig. 3a). Additionally, the environmental data at the planting locations were continuously monitored (Fig. 3b). Here, we evaluated prediction performance using three traits: flowering time (FT), PH, and yield (YLD) with distinct genetic architecture (Fig. S5, Tables S3, S5).

We initially used CERIS to search for an environmental index for each trait. Growing degree days (GDD) explained the largest amount of variation for FT, while the photothermal ratio (PTR) showed the highest correlation with PH and YLD (Fig. 3c, Figs S6, S7). These indexes were integrated into the two frameworks for predicting these traits across multiple environments. Overall, PEI framework exhibited superior prediction accuracy compared to that from the RN framework, with an increase of 10%, 13.9%, and 14.7% for FT, PH, and YLD (Fig. 3d–f, Table S4  Tables S6-S8). Consistent with observations from the CUBIC maize population, optimal estimators varied between two frameworks for different traits. For FT, MKRKHS yielded the highest prediction accuracy for RN framework, whereas G-BLUP outperformed the remaining estimators for PEI framework (Fig. 3d). When it comes to PH, RF outperformed other estimators for RN framework, while the RKHS was the best for PEI framework (Fig. 3e). In the case of YLD, the RF showed the highest accuracy for PEI framework (Fig. 3f). Overall, most estimators for the PEI framework demonstrated higher prediction accuracy than those from the RN framework (Fig. 3g–i).

### Prediction accuracy for multiple traits in an F_1_ willow population multi-environment trial

**Figure 4:**
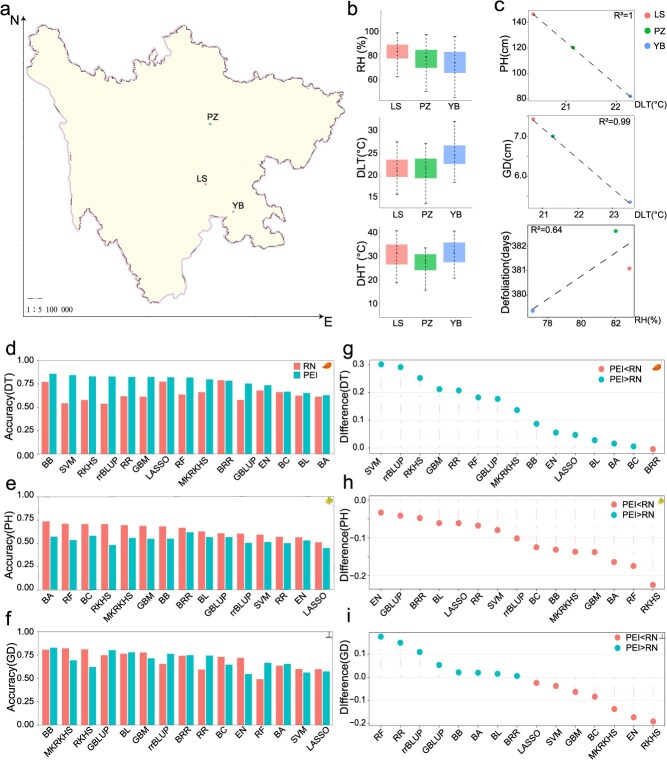
Prediction accuracy for multiple traits in an F1 willow multi-environment trial. (a) Geographic distribution of experimental locations for willow lines (Map Content Approval Number: GS (2017)1267); (b) Boxplot of the environmental datasets at the three locations. (c) Performance of the correlation between the mean phenotypes and environmental index within optimal time windows across diverse locations. (d-f) Prediction accuracy of the 15 estimators for the two frameworks on DT: Defoliation time; PH: Plant height; GD: Ground diameter; (g-i) Comparison of the prediction accuracy between PEI and RN frameworks across 15 estimators on DT, PH, and GD.

We finally benchmarked the prediction performance using an F_1_ willow population from a multi-environment trial. This population was derived from a complete diallelic cross among five elite male and five female outbred willow lines [49, 50]. A total of 501 F_1_ willow lines were obtained and phenotyped at three locations in Sichuan province, China. These locations ranged longitudinally from E 103°51′ at Leshan (LS) to E 104°36′ at Yibing (YB), and latitudinally from N 28°47′ at YB to N 29°37′ at LS (Fig. 4a). Temperature and relative humidity (RH) of the three planting locations were continuously monitored (Fig. 4b). Here, we tested the prediction performance using three traits: defoliation time (DT), PH, and ground diameter (GD), with varying genetic architecture (Fig. S8, Table S5).

Initially, we utilized CERIS to identify an environmental index corresponding to each trait. Daily lowest temperature (DLT) accounted for the greatest variation in DT and PH, while RH exhibited the strongest correlation with GD (Fig. 4c, Figs S9, S10, Table S6). These indexes were integrated into the two frameworks for predicting these traits across multiple environments. While PEI framework consistently outperformed RN across nearly all traits in the two inbred populations, its average accuracy was lower in this outbred population. Compared to the RN framework, PEI framework exhibited an average reduction in accuracy of 20% for PH and 1.6% for GD (Tables S10-S12). However, the PEI framework demonstrated an advantage in prediction accuracy, achieving 20.6% for DT. Furthermore, while most estimators favoured the RN framework over the PEI framework, the PEI framework showed modest prediction accuracy improvement of 9% for DT and 0.9% for GD, when using the best-performing estimators (Fig. 4d–f).

### Predicting phenotypes in novel environments

We finally evaluated the prediction performance of known genotypes in a novel environment. To do this, one of the tested environments was treated as unknown, and all phenotype measurements in that environment were set to ‘NA’. Prediction accuracy was calculated as the correlation between predicted and observed phenotypes at each site, and these correlations were then averaged across all sites. As the RN framework requires a minimum of three environments for linear regression, the F1 willow population was excluded from this analysis. To address differences in the number of environments between the maize and wheat inbred populations, we down sampled the wheat population to five planting locations.

**Figure 5:**
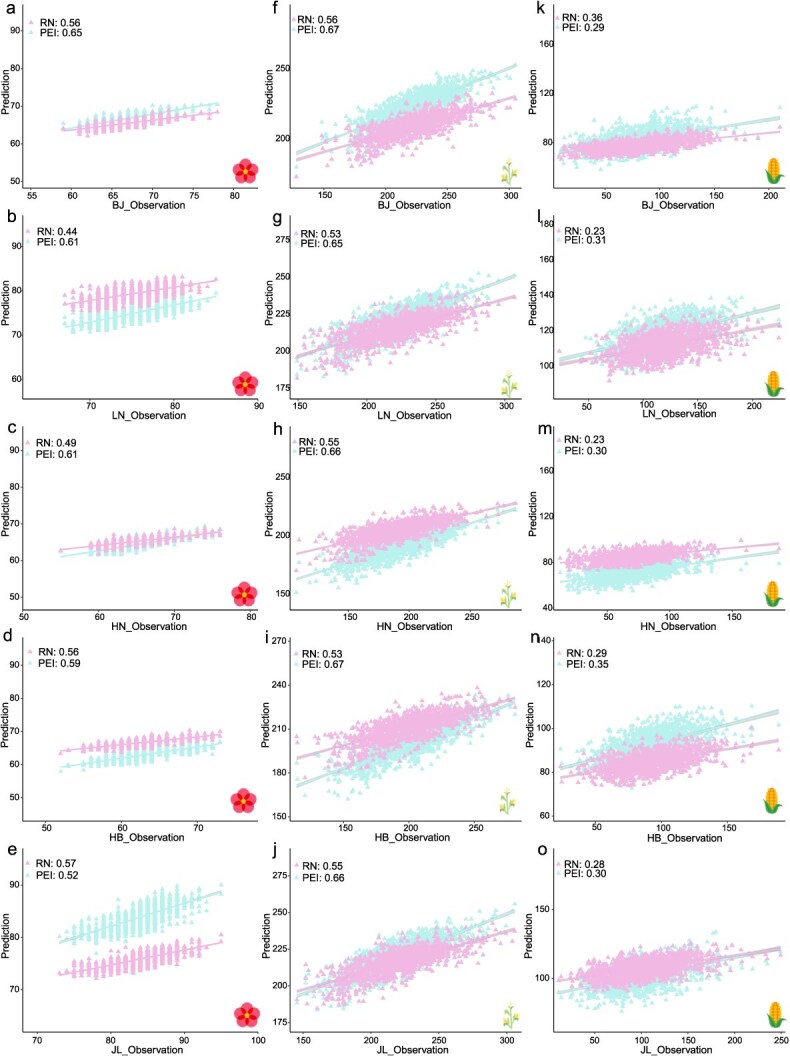
Predicting multiple traits in novel environments for a maize multi-environment trial. (a-e) The forecasting of DTA in the maize CUBIC METs for 5 locations (BJ, LN, HN, HB, JL); (b-j) The forecasting of PH in the maize CUBIC METs for 5 locations; (k-o) The forecasting of EW in the maize CUBIC METs for 5 locations.

For the maize CUBIC population, the PEI framework achieved higher predictive accuracy in 53.3% (8 out of 15) of predictions, spanning three traits across five locations, with an average improvement of 16.5% compared to the RN framework. Except for JL, the PEI framework delivered superior accuracy for DTA at all the remaining locations compared to the RN framework, with an average improvement of 13.7% in accuracy (Fig. 5a–e). For PH, the PEI framework consistently outperformed the RN framework at all five locations, with an average improvement of 21.7% in prediction accuracy (Fig. 5f–i). Regarding EW, the PEI framework exhibited higher accuracy for all locations except BJ, with an average improvement of 11.5% (Fig. 5k–o). Overall, the PEI framework demonstrated superior performance compared to the RN framework for predicting phenotypes in novel environments within the maize inbred populations (Fig. 5).

For the inbred wheat population, the PEI framework achieved higher predictive accuracy in 29.1% (9 out of 15) of predictions, encompassing three traits across five locations. However, the RN framework presented an average accuracy improvement of 2.9% over the PEI framework, driven by significantly better performance in the remaining locations (Fig. S11).

### Comparison of computing time and memory usage

**Figure 6:**
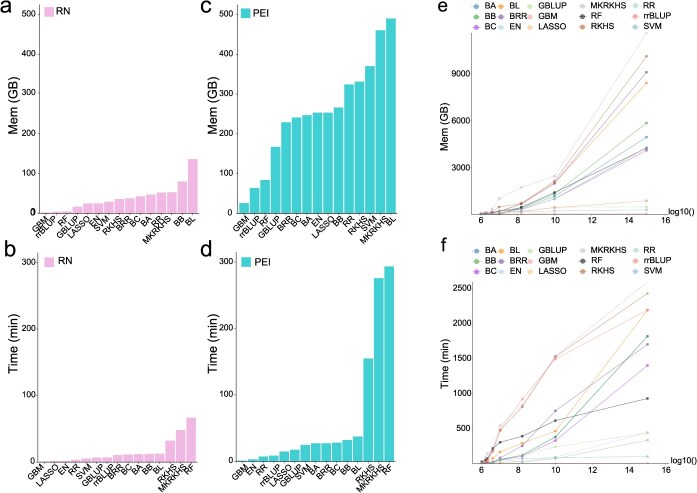
Comparison of computing time and memory usage. (a-b) Memory consumption and computing time of the 15 estimators for RN framework; (d-c) Memory consumption and computation time of the 15 estimators for PEI framework; (e-f) Scaling of memory consumption and computation time in relation to data dimension. Dimension (X-axis) was calculated as sample size multiplied by the number of genotype markers. They were transformed to -log10 scale to increase readability.

We finally compared the computing time and memory usage in relation to population size and parameter estimators. Since PEI framework involves the creation of super-matrices, whose dimension scales linearly with the number of environments, it requires more computational resources than RN framework when the number of environments increases. Here, we evaluated the computational cost under different population sizes by down and up sampling the CUBIC maize population for both frameworks and all the parametric estimators (Fig. 6a–d). For computational time, GBM has the shortest runtime, while the BL required the longest computing time. Meanwhile, GBM utilized the least amount of memory for both frameworks, while RF consumed the most. In addition, computing time and memory usage do not scale in a uniform manner across estimators (Fig. 6a–d). All the estimators exhibit an increase in computation time and memory usage as the data dimension increases (Fig. 6e and f). Semi-parametric estimators (e.g. RKHS and MKRKHS) showed the greatest increase in computing time and memory usage, while non-parametric estimators (e.g. GBM and SVM) remained relatively stable as the data dimensionality increases (Fig. 6e and f). Running PEI on a standard workstation (e.g. 16-core CPU, 64 GB RAM) showed that when dimension increases from 7 to 8, the computational cost increased substantially while prediction accuracy remained low (Fig. S12). Here, ‘dimension’ refers to the order of magnitude (base 10) of the product of population size and number of markers. For example, 100 lines × 10 000 markers = 1 000 000 = 10 [6], giving a dimension of 6. When dimension increases from 7 to 8, the gain in computational cost is high, but the accuracy is low. Therefore, users are advised to consider alternative estimators under this scenario.

## Discussion

In this study, we presented a novel framework to model complex traits variation in METs by directly accounting for the combined effects from genotype, environment, and their interactions. By incorporating a super matrix that integrates environmental index effects and their interactions with the polygenic background, this approach significantly improved our capacity to model complex traits in METs (Fig. 1b). To mitigate the computational challenges associated with handling a massive number of markers, we adopted two solutions. One involved marker pruning based on linkage disequilibrium [51]. The other leveraged machine learning and deep learning approaches to maintain prediction accuracy without significantly increasing computational burden. These approaches allowed our framework to match the performance of existing estimators. Furthermore, it did so without incurring an excessive computational load.

Through benchmarking analysis with multiple traits across diverse populations, we observed that the PEI framework significantly outperforms the RN framework in two inbred populations (Figs 2–4). However, in the F_1_ willow population, the PEI framework showed superior predictive ability only for DT. This is likely because complex interactions associated with various traits in a hybrid population (Fig. 4). Previous studies in hybrid populations have revealed that epistatic variance is the major component of phenotypic variance [52, 53]. We therefore tested whether RN framework is better at capturing such non-additive effects using a yeast growth dataset. This dataset contains 4000 genotype and phenotype measurements of yeast growth on 20 different medium, and four early studies have mapped out the additive QTLs, and epistatic network in 20 environments [52] (Supplementary Notes). We found that the slope parameter from the RN framework was correlation with the degree of epistatic interaction, whereas the intercept parameter did not (Fig. S13). This suggests that the RN slope parameter could capture epistatic interactions indirectly. In contrast, the PEI framework may be less suited to model the complex genetic architecture in hybrid populations, where epistasis contributes substantially to phenotypic variation. Our findings highlight the profound influence of genetic architecture and population structures on prediction performance. We, thus, recommend performing comprehensive comparisons to select the optimal strategy in future applications.

It should be noted that predicting phenotypes in novel environments remains challenging due to the complex gene–environment interactions. This challenge is particularly pronounced for METs that span a wide range of environments. In the inbred wheat METs analysed here, the PEI framework performed poorly for IH10, likely because IH10 differs significantly from the other planting locations (Fig. 3 and Figs S8, S14). Distance-based analyses revealed that IH10 exhibits both the largest minimum distance and the greatest overall dissimilarity relative to the remaining environments, indicating a weak environmental continuity (Fig. S14). This discontinuity reduces prediction performance in the PEI framework, which assumes the pattern of genotype by environment interaction is similar across tested sites. Additional analysis showed that prediction accuracy is consistently reliable when an environment’s Youden’s index to the training set is ≤3.75. Above this threshold, prediction accuracy drops substantially (Supplementary Notes, Fig. S14). Thus, the environmental outlier (IH10) reduces prediction accuracy in the PEI framework due to weak environmental continuity (Fig. S14). Increasing the environmental continuity of planting locations might capture the complex interaction pattern between a larger range of ecological factors and genotype, but it would certainly increase the costs of METs [54].

Finally, both RN and PEI frameworks include genetic interaction with the single most relevant environmental index, which introduces two potential caveats. Model performance might be sensitive to the selected environment index and including multiple indexes might improve the model performance. Additional analysis shown that compared to the RN framework, PEI framework exhibited substantially low sensitivity to environmental variation (Fig. S15). These indicate that the PEI is less sensitive than the RN to selection of environmental index, which yielded more robust performance. Furthermore, we demonstrated that while model complexity and computational time increased significantly with including more environment index, prediction accuracy improved in only a few trait and population combinations (Fig. S16). This is because most of the phenotypic variance is captured by the first environmental index, making the gain from adding further index marginal (Fig. S17). In addition, we attempted to benchmark our implementation with existing frameworks with publicly available code. In contrast to our framework, where CERIS always determines the optimal factors, GEFormer used all available environmental data as input, allowing the framework to integrate it independently, which to some extent avoids bias. Despite this, we found no or only marginal gain in prediction accuracy for various trait and site combinations, suggesting our PEI framework is relatively robust (Table S13 and Figs S18, S19). However, our attempt to benchmark against a recently published framework. AutoGS [55], failed due to unstable release of the software going through constant updates. A future comparison will be essential for a fully comprehensive evaluation once a stable, well-documented version of the software is released.

In summary, we introduce a unified framework, PEI, that directly models genotype-by-environment interactions through integrating genotypes and ECs. We implemented 15 estimators, including parametric, non-parametric, and machine learning approaches, to improve prediction accuracy. Alongside the classical RN framework and recently developed deep learning approaches, we conducted benchmarking analysis using three distinct populations with various population structures and three traits with variable genetic architectures. Additionally, we released an R package, MMGS available on GitHub (https://github.com/Ryougi-yukiro/MMGS). Our framework and software package could facilitate multi-environment genomic prediction, improve breeding efficiency, and offer deeper insights into modelling the relationship between genotype and environment.

## Materials and methods

### Datasets used for genomic prediction

Three datasets, reflecting diverse population sizes and structures, were utilized in this investigation. The first dataset is CUBIC maize population, including 1404 inbred maize lines, and their performance was assessed at five locations within China’s primary maize production zone. This zone exhibits longitudinal variation ranging from E 114°01′ at HN to E 125°18′ at JL and latitudinal variation from N 43°42′ at JL to N 35°27′ at HN [46]. In essence, this population was derived from 24 elite varieties, representing four divergent heterotic groups, through cycles of random mating, selection, and inbreeding. Three environmental variables, specifically DHT and DLT, along with daily length (DL), were downloaded from Zan *et al*. Genotyping data of the CUBIC maize population were a subset of the maize variants file uploaded, then we filtered these data through Plink 1.9 [56]. A total of 28 973 SNPs among 1007 individuals from 5 locations were used for the next analysis [47].

The second dataset, the inbred wheat population downloaded from the CIMMYT [2], consists of 287 inbred lines planted in India, Pakistan, Nepal, Bangladesh, Iran, Egypt, Sudan, and Mexico. In each location, plants were grown in plots with an α-lattice design with two replications. Four environmental variables, specifically GDD, PTR, and photothermal time (PTT), along with DL, were downloaded in Li *et al*. [22]. Each genotype was planted in a single-row plot of 8 to 15 plants. The wheat population was genotyped with an Illumina iSelect 90K SNP assay for 26 814 SNPs [48]. The HapMap file was formatted into plink format through Tassel [57].

The third dataset, an F1 willow population, consisted of 501 F_1_ lines planted in YB, Pengzhou (PZ), and LS. Each genotype was planted in a single-row plot of a single plant. Three environmental variables, specifically DHT and DLT, along with RH were collected from three plots. Whole genome sequencing data of the willow population were processed by GATK v3.8, and over 12 million willow SNPs with missing rate higher than 20% and minor allele frequency less than 1% were obtained. Then all variants were imputed by Beagle [58] following the default parameters.

### DNA sequencing and environmental factor acquisition

Total DNA was collected and extracted from fresh leaves of *Salix suchowensis* using the CTAB method. Libraries were constructed and sequencing were performed by Novogene using Illumina NovaSeq platform. Raw reads were quality controlled through fastp [59]. Then, we used bwa-mem2 to map raw reads to the reference genome. The GATK pipeline was utilized for SNP calling [60]. SNPs were subsequently filtered using VCFtools to retain only bi-allelic variants with call rates above 90%, and minor allele frequency greater than 0.05 [61].

To obtain the environmental factors, continuous monitoring was carried out using micro weather stations, built by the Beijing Aozuo Ecological Instrument Co., set up in the field from May to November each year. The system consists of various types of meteorological sensors, data acquisition system, power supply system and so on. It measures wind speed and direction (10m, 5m, 2m), temperature and RH (10m, 5m, 2m), rainfall, atmospheric pressure, gross radiation, net radiation, soil moisture, soil temperature and electrical conductivity at regular intervals and automatically according to the measurement intervals set by the user. The monitoring data excludes the days missed by the machine and the final environmental data is used for the time for which continuous records exist at all three locations.

### CERIS for searching environmental index

The CERIS algorithm used for identifying the environmental indexes had been described in the previous study [22]. For the tested populations, we began by calculating the mean values for all individuals across various phenotypes, which were then used as the horizontal coordinates. In addition, we conducted sliding window calculations, utilizing both environmental and phenotypic data over a continuous observation period with equal-sized bins. This procedure allowed us to identify the most appropriate start and end times for corresponding traits.

To validate the relevance of this approach, we performed a regression analysis. This involved using environmental data from various sites within the identified window as explanatory variable, while the mean trait values from these sites were used as the response variable. The environmental factor that yielded the highest *R*^2^ value was considered as the most relevant factor and was selected for further analysis. This approach ensured that the identified window and environmental factor were robustly linked to the phenotypic traits under consideration.

### Reaction norm framework

Construction of the RN framework relies heavily on previous research [18, 22]. It quantifies individual response to changes in environment factor as a unique intercept and slope using linear regression. For each individual $i$, a linear regression was performed with the environmental index detected in previous section as the explanatory variable *x* and the phenotypes in environment *j* as response variable ${y}_{ij}$.


(1) \begin{equation*} {y}_{ij}={k}_ix+{b}_i+{e}_{ij} \end{equation*}

${y}_{ij}$ represents the phenotypes of $i$ individual in $j$ environment, $x$ is the is the environmental-index value of $j$ environment centred by the average environmental-index values across the environments, and ${e}_{ij}$ is the residual. ${k}_i$ and ${b}_i$ is the slope and intercept for individual *i*, representing two unique traits of environmental response.

Genomic prediction in the RN framework using rr-BLUP was performed as:


(2) \begin{equation*} y=\mu + Zg+\varepsilon \end{equation*}

where $y$ is the phenotypic values (i.e. either intercept or slope derived for equation (1)), $\mu$ is the overall mean. $g$ is a vector of random genetic effects, and $\varepsilon$ is a vector of residuals variance. $Z$ are incidence matrices. The variance of the random effects $g$ is $\operatorname{var}(g)=G{\sigma}_g^2$, where G is the genomic relationship matrix and ${\sigma}_g^2$ is the additive genetic variance. Predicted phenotype for a specific individual was obtained by plugging in estimated ${k}_i$ and ${b}_i$ from equation (2) back to equation (1).

### Polygenic-environment-interaction framework

We modelled the phenotype measured in multiple environments using mixed linear model.

Let ${y}_{ij}$be the phenotype of *i*th *(i = 1, 2, 3,. . .,n)* individual measure at *j*th *(j = 1, 2, 3,. . ., m)* environment. In a specific environment *j,*  ${y}_{ij}$ (length *n*) is affected by a linear combination of random marker effects ${u}_j$ and residual error ${e}_j$. The length of ${u}_j$ is equal to the number of markers, *p*, and the random marker effects are assumed identically and independently distributed (iid) and normal, so that ${u}_j$ *~ N* (0, *I*${\sigma}_u^2$). ${e}_j$ is the residual error *e ~ N* (0, *I*${\sigma}_e^2$). Thus, a linear mixed model for this environment is:


(3) \begin{equation*} {y}_j={\mu}_j+M{u}_j+{e}_j \end{equation*}

whereby *μ* is a population mean, and matrix *M* is the additive genotype matrix coded as 0, 1, and 2, with *n* by *p* dimension. *n* is the number of individuals and *p* is the number of markers


(4) \begin{equation*} M=\left(\begin{array}{@{}cccc@{}}1& 0& \cdots & 0\\{}2& 1& \cdots & 2\\{}\vdots & \vdots & \ddots & \vdots \\{}0& 2& \cdots & 1\end{array}\right)\kern0.5em \end{equation*}

An equivalent G-BLUP model can be obtained by define an individual random effect ${\mathrm{\alpha}}_j$ (length *n*). such that ${\mathrm{\alpha}}_j$  *=*  ${Z}_j{u}_j$, and we thereby have:


(5) \begin{equation*} {y}_j={\mu}_j+{\alpha}_j+{e}_j \end{equation*}

where *V(*$\mathrm{\alpha}$*) = G*${\sigma}_u^2$. Here G is the genomic relationship matrix, and we have *G=*${Z}_j{Z}_j$*′.*

Extending this univariant mixed model to multiple environments, we could construct a multi environment phenotype vector *y* by stacking up the phenotype vector, ${y}_1$ to ${y}_m$, measured from each environment.


(6) \begin{equation*} {y}_j=\left(\begin{array}{@{}c@{}}{y}_{1j}\\{}{y}_{2j}\\{}\vdots \\{}{y}_{nj}\end{array}\right),\kern1em y=\left(\begin{array}{@{}c@{}}{y}_{11}\\{}{y}_{21}\\{}\vdots \\{}{y}_{nm}\end{array}\right) \end{equation*}

Assuming there is no genotype by environment interaction, we have


(7) \begin{equation*} y=\mathrm{\mu} +E+ Bu+e \end{equation*}

whereby *E* is an environment specific environmental effect vector (length *n × m*), *u* and *e* is the stacked marker effects and residual errors, and *B* is a super genotype matrix with *n × m* rows and *p* columns.


(8) \begin{equation*} E=\left(\begin{array}{@{}c@{}}{E}_1\\{}{E}_2\\{}\vdots \\{}{E}_m\end{array}\right) \end{equation*}

${E}_j$ is a vector of length *n* representing the most relevant environmental effects, which is obtained using the CERIS algorithms as described above.


(9) \begin{align*} B={I}_m\otimes M=\left(\begin{array}{@{}cccc@{}}M& 0& \cdots & 0\\{}0& M& \cdots & 0\\{}\vdots & \vdots & \ddots & \vdots \\{}0& 0& \cdots & {M}_m\end{array}\right) \end{align*}

${I}_m$ is an identity matrix with *m* dimension, which corresponds to the number of environments. $B$is a diagonal matrix with genotype matrix, excluding *E*.


(10) \begin{equation*} u=\left(\begin{array}{@{}c@{}}{u}_{11}\\{}{u}_{21}\\{}\vdots \\{}{u}_{pm}\end{array}\right) \end{equation*}

(11) \begin{equation*} e=\left(\begin{array}{@{}c@{}}{e}_{11}\\{}{e}_{21}\\{}\vdots \\{}{e}_{nm}\end{array}\right) \end{equation*}

An equivalent G-BLUP model can be obtained by define an individual random effect *α* (length *n × m*). such that


(12) \begin{equation*} \mathrm{\alpha} =\left(\begin{array}{@{}c@{}}{\mathrm{\alpha}}_1\\{}{\mathrm{\alpha}}_2\\{}\vdots \\{}{\mathrm{\alpha}}_m\end{array}\right) \end{equation*}

and we thereby have:


(13) \begin{equation*} y=\mathrm{\mu} +E+ Z\alpha +e \end{equation*}

where *V(*$\mathrm{\alpha}$*) = K*${\sigma}_u^2$ . Here, *K* = ${I}_m\otimes \boldsymbol{G}$ is the relationship matrix. And this model can be solved as a univariant G-BLUP model.

To model the interaction between the environment and polygenic background, we constructed a genotype by environment interaction matrix at environment *j* with the following equation:


(14) \begin{equation*} {M}_j^{\mathrm{inter}}={E}_jM=\left(\begin{array}{@{}cccc@{}}1{E}_j& 0{E}_j& \cdots & 0{E}_j\\{}2{E}_j& 1{E}_j& \cdots & 2{E}_j\\{}\vdots & \vdots & \ddots & \vdots \\{}0{E}_j& 2{E}_j& \cdots & 1{E}_j\end{array}\right) \end{equation*}

We then combined the matrix $M$ and ${M}_j^{\mathrm{inter}}$ as a new genotype matrix ${M}_j^{\mathrm{all}}$


(15) \begin{equation*} {M}_j^{\mathrm{all}}=\big[M\{M}_j^{\mathrm{inter}}\ \big] \end{equation*}

This is equivalent to double the number of makers by including a pseudo marker vector representing the interaction from each of these markers and the environment index.

Similarly, a multiple environment super genome matrix could be constructed.


(16) \begin{equation*} {B}_{\mathrm{all}}=\left(\begin{array}{@{}cccc@{}}{M}_1^{\mathrm{all}}& 0& \cdots & 0\\{}0& {M}_2^{\mathrm{all}}& \cdots & 0\\{}\vdots & \vdots & \ddots & \vdots \\{}0& 0& \cdots & {M}_m^{\mathrm{all}}\end{array}\right) \end{equation*}

where ${B}_{\mathrm{all}}$ is a diagonal matrix with new genotype matrix ${M}_j^{\mathrm{all}}$ combined with ${E}_j$.

Therefore, by plugging this back to model (7) and (13), we could make predictions for multi-environment phenotype in the mixed linear model framework.

### Benchmark against GEFormer framework

We followed the original design of GEFormer and applied it to our datasets. Hyperparameter selection was performed using the Optuna Bayesian optimization library, with a search conducted on the training set for various traits of three populations to obtain optimal configuration. All GEFormer analyses were implemented in Python 3.9.19 using PyTorch. A complete description of training hyperparameters across various datasets in the supplementary tables (Table S14).

### Cross-validation schemes

PEI performance predicted phenotypes encompass two scenarios: (i) forecasting the performance of untested genotypes in tested environments, and (ii) predicting the performance of tested genotypes in untested environments. Unlike traditional joint regression analyses, such as the RN framework, the PEI framework relies on genotypic data and exploits potential relationships between tested genotypes and phenotypes to predict the phenotypes of untested genotypes. Additionally, by analysing correlations between environments and environmental indices, the method forecasts the performance of individuals without phenotypic data in future environments, drawing upon phenotypic data from existing environments.

In the first scenario, a leave-one-half-of-genotypes-out cross-validation was performed. (i) The $n$ genotypes were randomly divided into two equal groups: tested genotypes and untested genotypes. (ii) The environmental index was identified using CERIS, based on the environmental means derived from the tested genotypes. (iii) Model (13) was then used to make predictions for the untested genotypes. Genomic prediction was then executed using various estimators to forecast the phenotypes of each untested genotype.

In the second scenario, leave-one-environment-out cross-validation was conducted. (i) Each environment was sequentially treated as the untested environment, with the remaining environments serving as the tested environments. (ii) The environmental index was identified using CERIS, based on the environmental means of the tested genotypes in the tested environments. (iii) With the obtained environmental index, a genotype by environment interaction matrix was built using model (16). (iv) Model (13) was then used to make predictions for the untested genotypes.

Prediction accuracy was assessed as the correlation between observed and predicted values. The predicted values were derived from a framework built using the training set of tested genotypes in tested environments. Accuracy was evaluated both across all environments and at the individual environment level. During genotype sampling, the average prediction accuracy across five replications with 10-fold was calculated. A representative run is provided in the figure for illustration.

### Description of modelling and parameter estimators

We employed the *sf* [62] package to generate a map of China and the *rworldmap* [63] package to create a world map. Consider that forecasting estimators are divided into parametric and non-parametric estimators and that the principles of different parametric estimators vary. Most of them are based on additive models, and their accuracies may be different because they vary in their assumptions and algorithms with respect to the variances of complex traits. Incorporating non-additive effects or multiple variates, the general estimators can be extended (Fig. S20) [15, 64]. Various estimators were implemented by leveraging parameters from R packages [42, 43, 65, 65–67]. Finally, we illustrated the average prediction accuracy of 15 distinct estimators, encompassing parametric, semi-parametric, and non-parametric approaches, for both frameworks using ggplot2 [68], with adjustments made in Adobe Illustrator.

### Heritability estimation

The kinship heritability of each trait was calculated as follows:


(17) \begin{equation*} H=\frac{V_g}{V_g+\frac{V_{ge}}{L}+\frac{V_e}{RL}} \end{equation*}

where ${V}_g$ the epigenetic variance, ${V}_{ge}$ represents the gene–environment interaction variance, *L* indicates the number of environments, ${V}_e$ signifies the environmental variance, and R was the number of replicates.

## Supplementary Material

Web_Material_uhag035

## Data Availability

The test datasets, including genotypic, phenotypic, and environmental data, can be accessed at this GitHub repository (https://github.com/Ryougi-yukiro/MMGS-bench). Additionally, the benchmark codes have been uploaded to this repository. For quick installation of the developed R package, kindly refer to the GitHub repository: https://github.com/Ryougi-yukiro/MMGS. This repository not only facilitates the installation of the R package but also provides example codes tailored for quick start. Detailed instructions can be found in the R documents and accompanying tutorial (https://multiplemethodgs.gitbook.io/MMGS_tutorial_v1).
