# Supplementary material for: MMGS: a novel genomic prediction framework to integrate genotype, environment and their interactions for multi-environment breeding trials
Source: Hortic Res. 2026 Mar 16;13(5):uhag035. doi: 10.1093/hr/uhag035 (PMC13150855; doi:10.1093/hr/uhag035)
Supplement: Web_Material_uhag035 [file web_material_uhag035.zip › Suplementary_Figures_tables-20251218zyj.docx]

**Supplementary Figures**


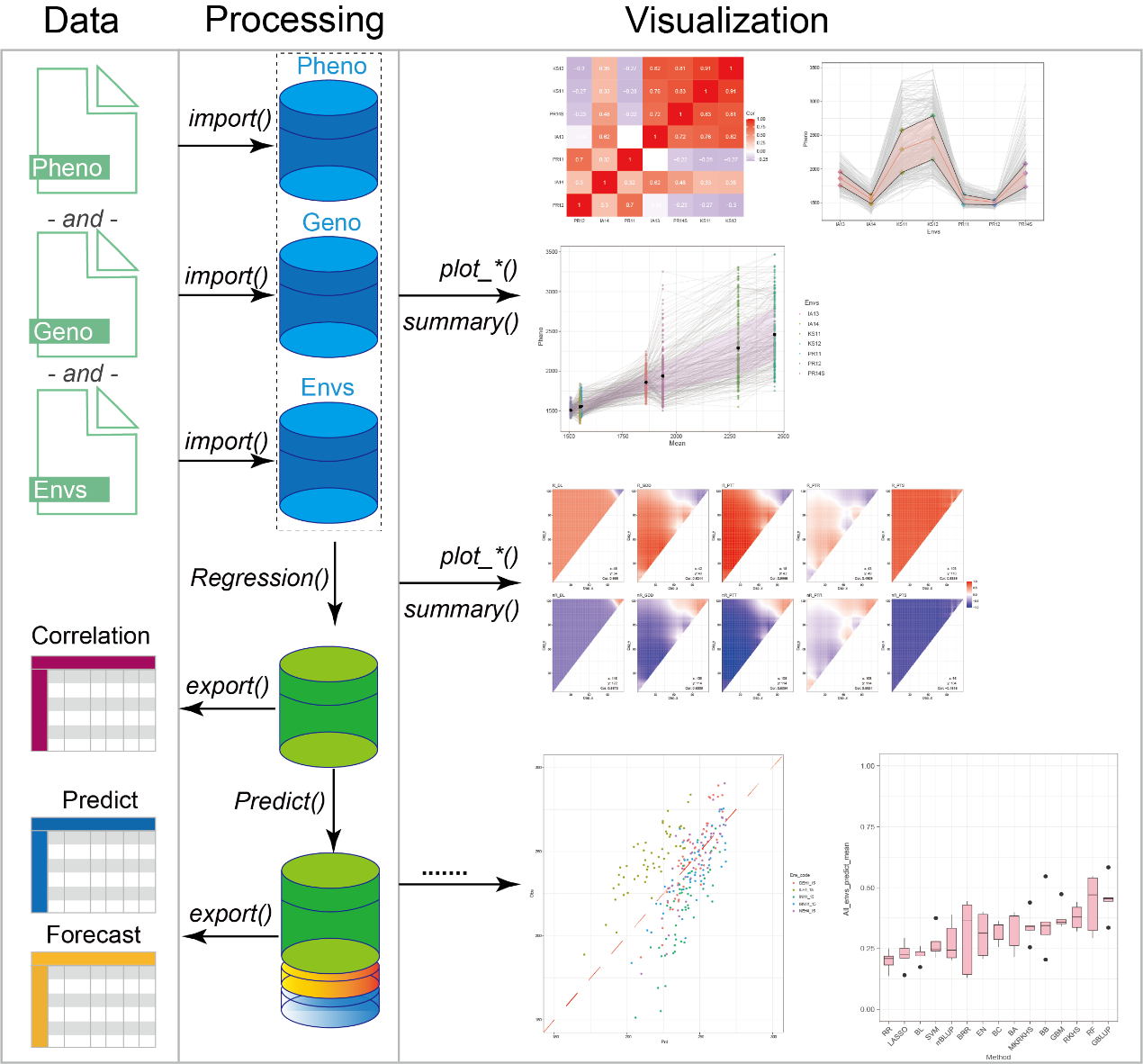


**Fig S1.** **Flowchart of the Multiple-environmental Multiple-methods Genomic Selection (MMGS) pipeline.**


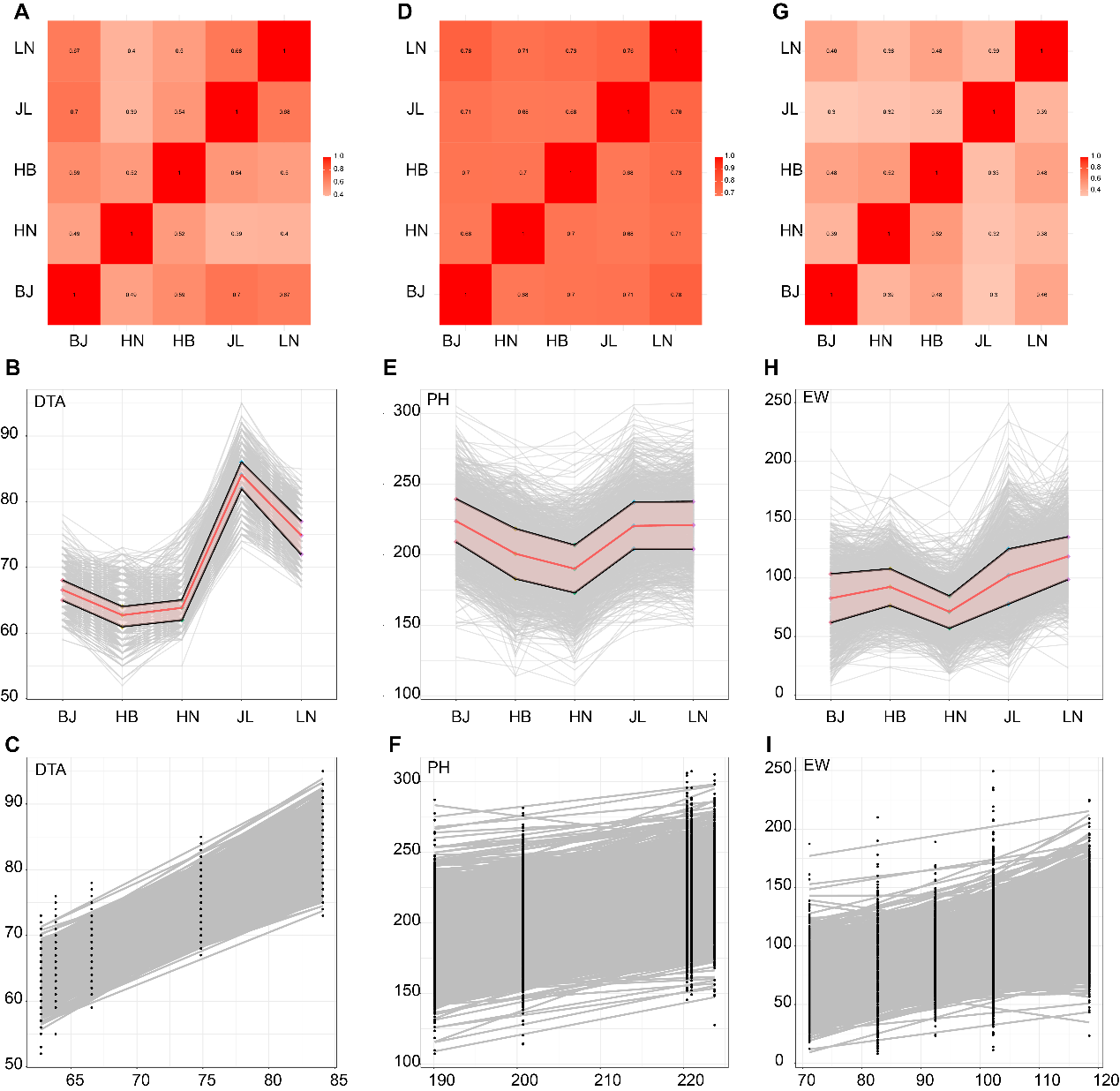


**Fig S2.** **Summary of three maize traits at 5 locations.** (A) Correlation of DTA among 5 locations. (A) Correlation of DTA among 5 locations. (B) Line chart of DTA across 5 locations. The red line represents the mean DTA, and the black lines represent the quartiles. The y-axis is in days. (C) Regression of DTA on within site DTA mean. The x-axis represents the mean DTA for each location. (D) Correlation of PH among 5 locations. (E) Line chart of PH. The red line represents the mean PH, and the black lines represent the quartiles. The y-axis is in cm. (F) Regression of PH on within site PH mean. The x-axis represents the mean PH for each location. (G) Correlation of the EW trait among 5 locations. (H) Line chart of EW. The red line represents the mean EW, and the black lines represent the quartiles. The y-axis is in kg. (I) Regression of EW on within site EW mean. The x-axis represents the mean EW at each location.


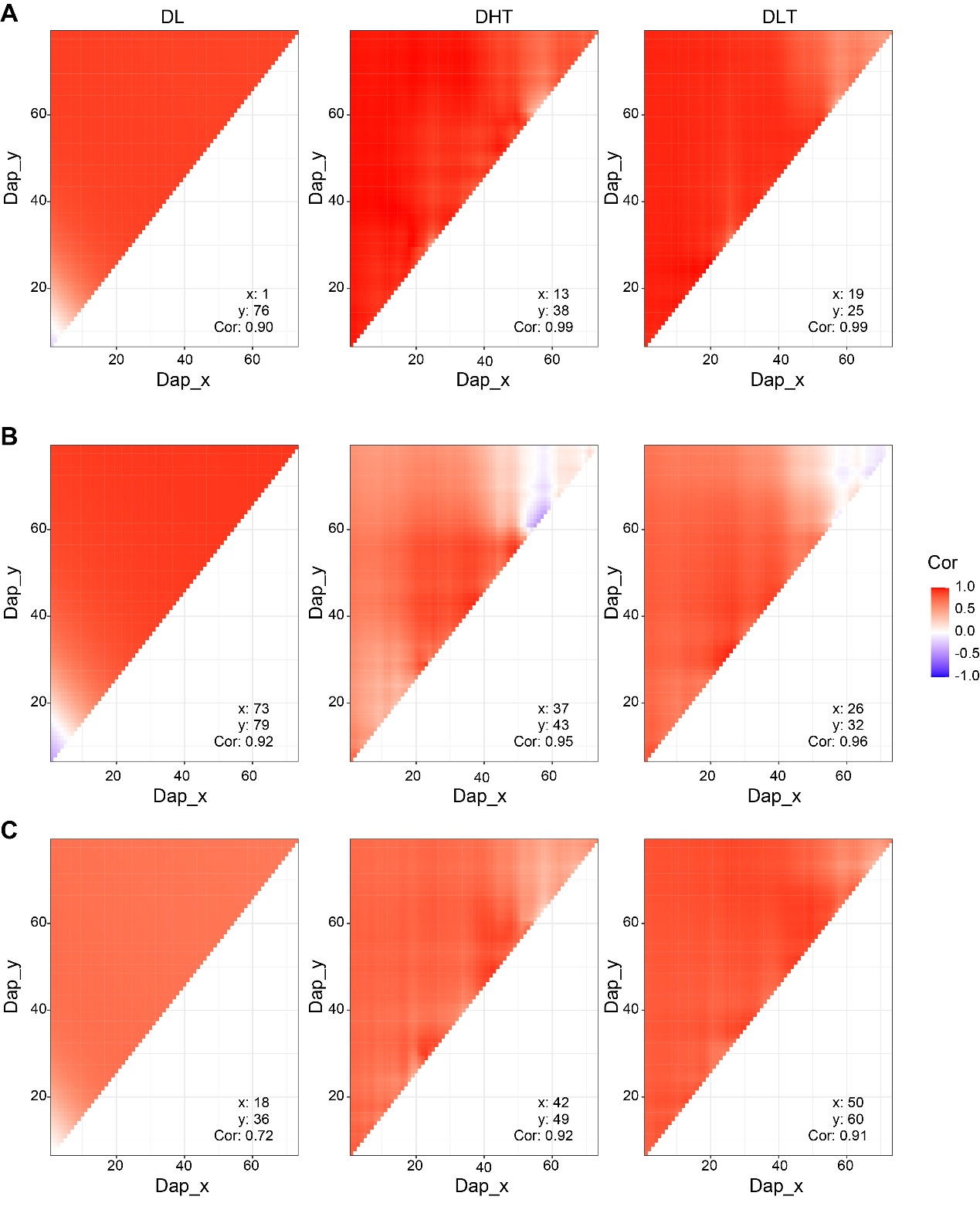


**Fig S3.** **Search for the most influential environmental index on maize traits using the CERIS algorithm.** The algorithm scans daily environmental data across the entire growth period to identify the time window (start and end day) with the strongest absolute correlation to the phenotypic trait mean across locations. The correlation for each window is calculated using Pearson's correlation. The three environmental indexes tested are: DL (day length), DHT (daily highest temperature), and DLT (daily lowest temperature). (A) Days to anthesis (DTA). (B) Plant height (PH). (C) Ear weight (EW).


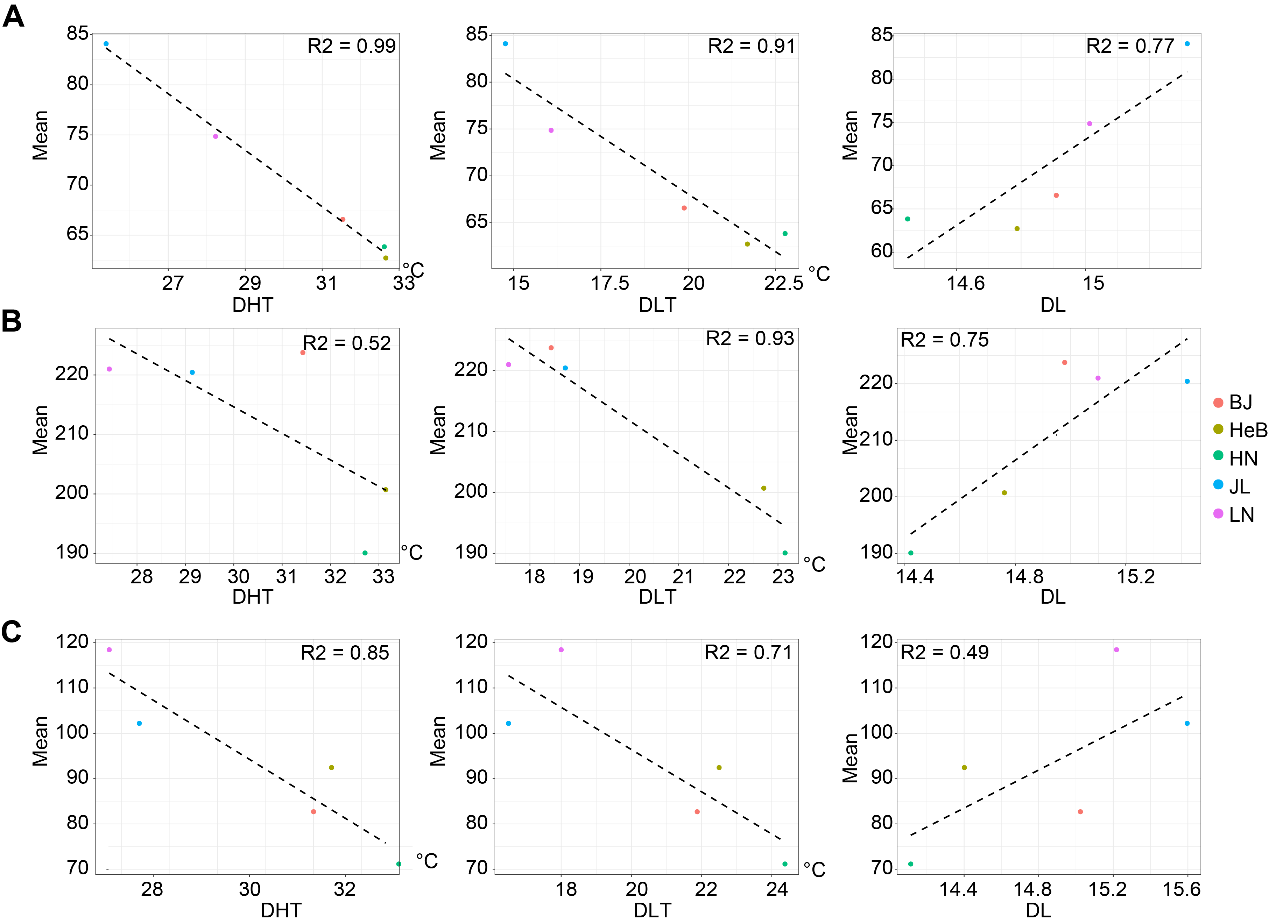


**Fig S4.** **Regression of maize traits on the environmental index identified by CERIS.** The x-axis shows the mean value of the environmental index calculated for the identified window, while the y-axis shows the mean trait value for that same period. The R² value indicates the goodness of fit for each regression. (A) Days to anthesis (DTA). (B) Plant height (PH). (C) Ear weight (EW).


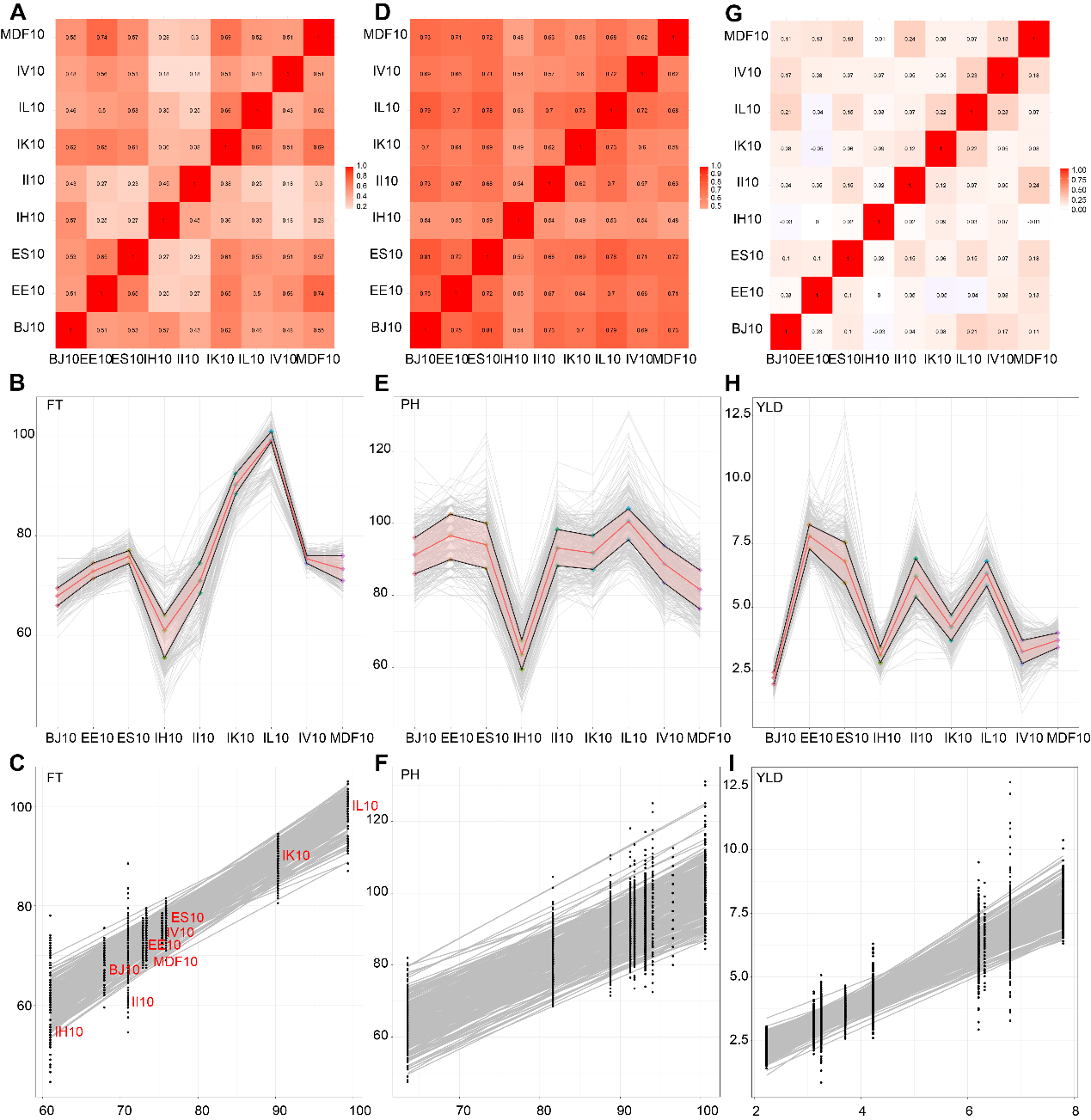


**Fig S5.** **Summary of three wheat traits across nine locations.** (A) Correlation of FT among locations. (B) Line chart of FT. The red line represents the mean FT, and the black lines represent the quartiles. The y-axis is in days. (C) Regression of FT on within site FT mean. The x-axis represents the mean FT for each location. (D) Correlation of PH among locations. (E) Line chart of PH. The red line represents the mean PH, and the black lines represent the quartiles. The y-axis is in cm. (F) Regression of the PH trait. The x-axis represents the mean PH for each location. (G) Correlation of YLD among locations. (H) Line chart of YLD. The red line represents the mean YLD, and the black lines represent the quartiles. The y-axis is in grams (g). (I) Regression of the YLD trait. The x-axis represents the mean YLD for each location.


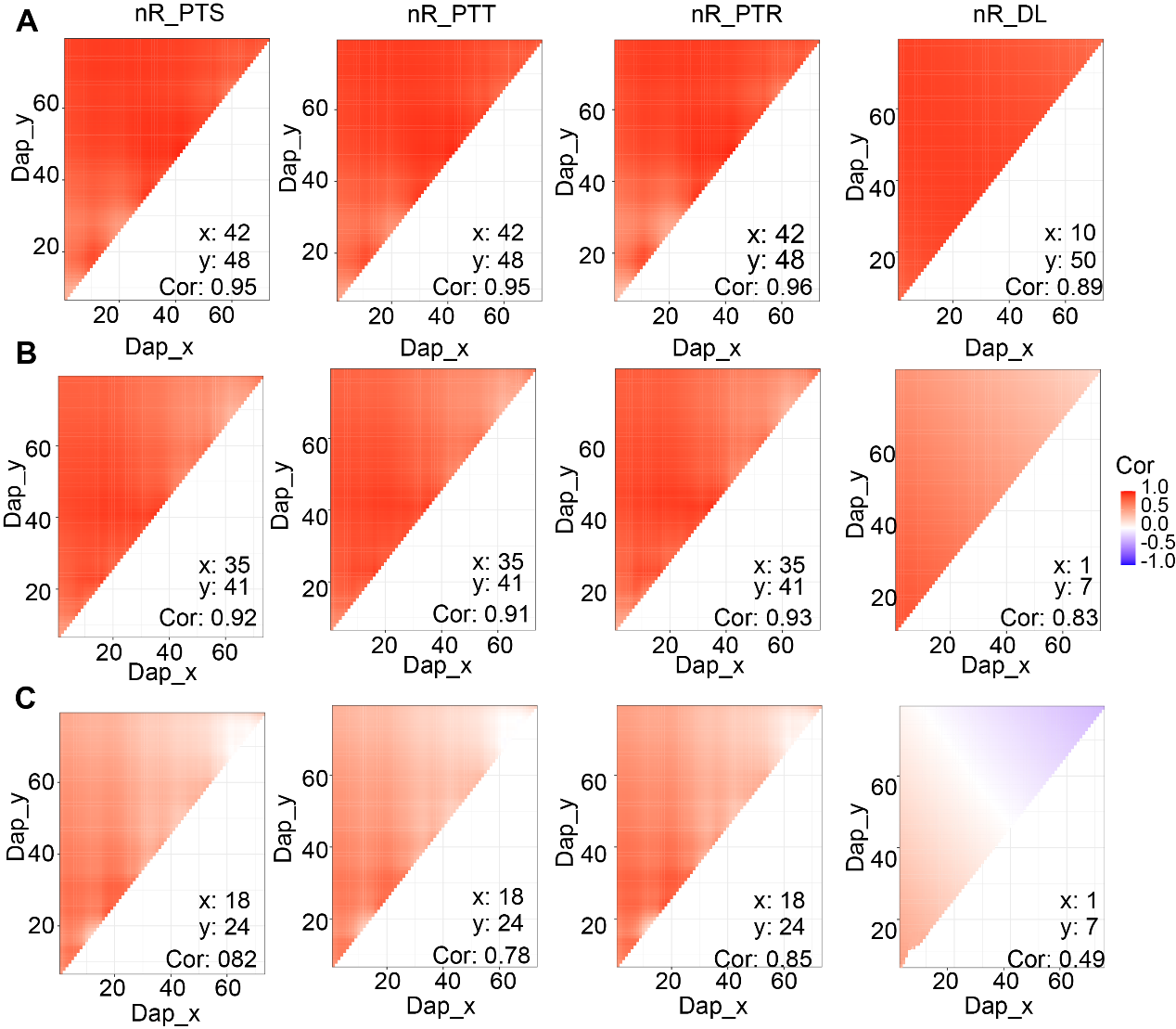


**Fig S6.** **Search for environmental indexes influencing wheat traits using the CERIS algorithm.** The algorithm scans four daily environmental indexes—day length (DL), photothermal time (PTT), and photothermal ratio (PTR)—across the entire growth period to identify the time window (start and end day) with the strongest absolute correlation to the phenotypic trait mean across locations. The correlation for each potential window is calculated using Pearson correlation. (A) Flowering time (FT). (B) Plant height (PH). (C) Yield (YLD).


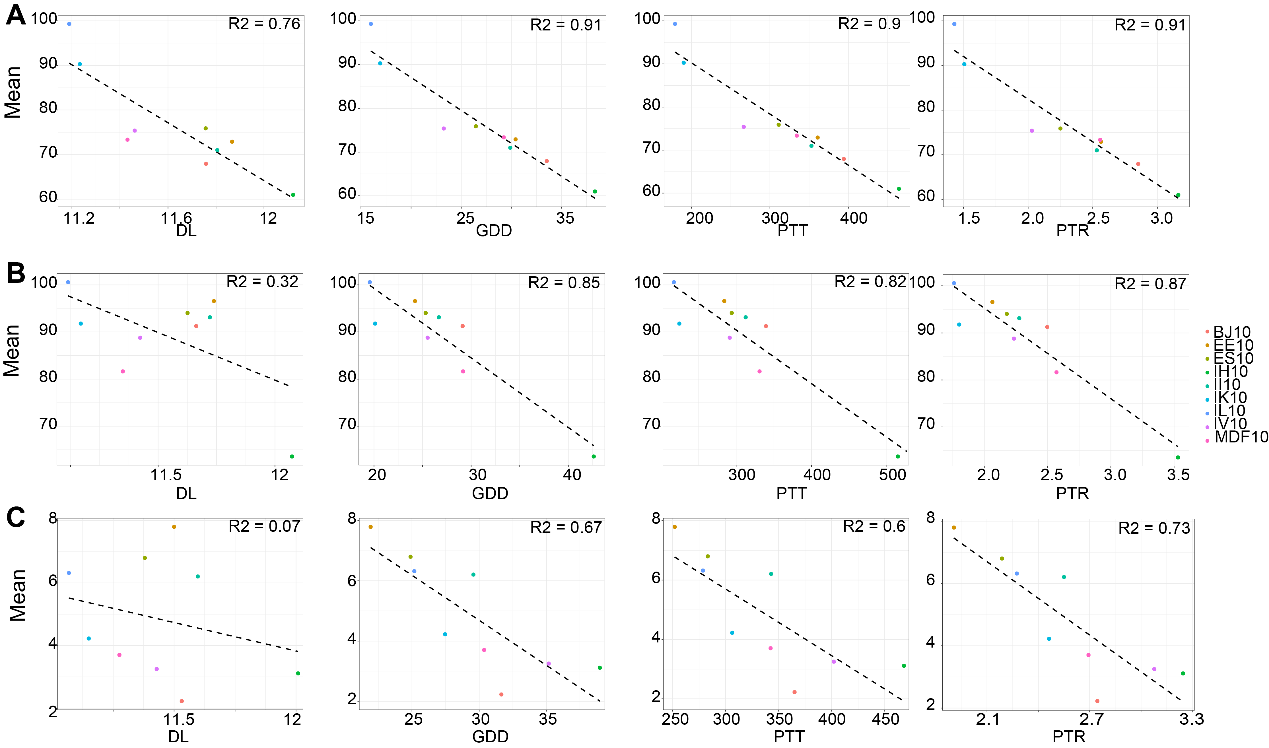


**Fig S7. Regression of wheat traits on their identified environmental index.** For each trait, the x-axis shows the mean value of the environmental index (e.g., PTR, PTT) calculated for its strongest correlated window, while the y-axis shows the mean trait value for that same period. The R² value indicates the goodness of fit for each regression. (A) Flowering Time (FT). (B) Plant Height (PH). (C) Yield (YLD).


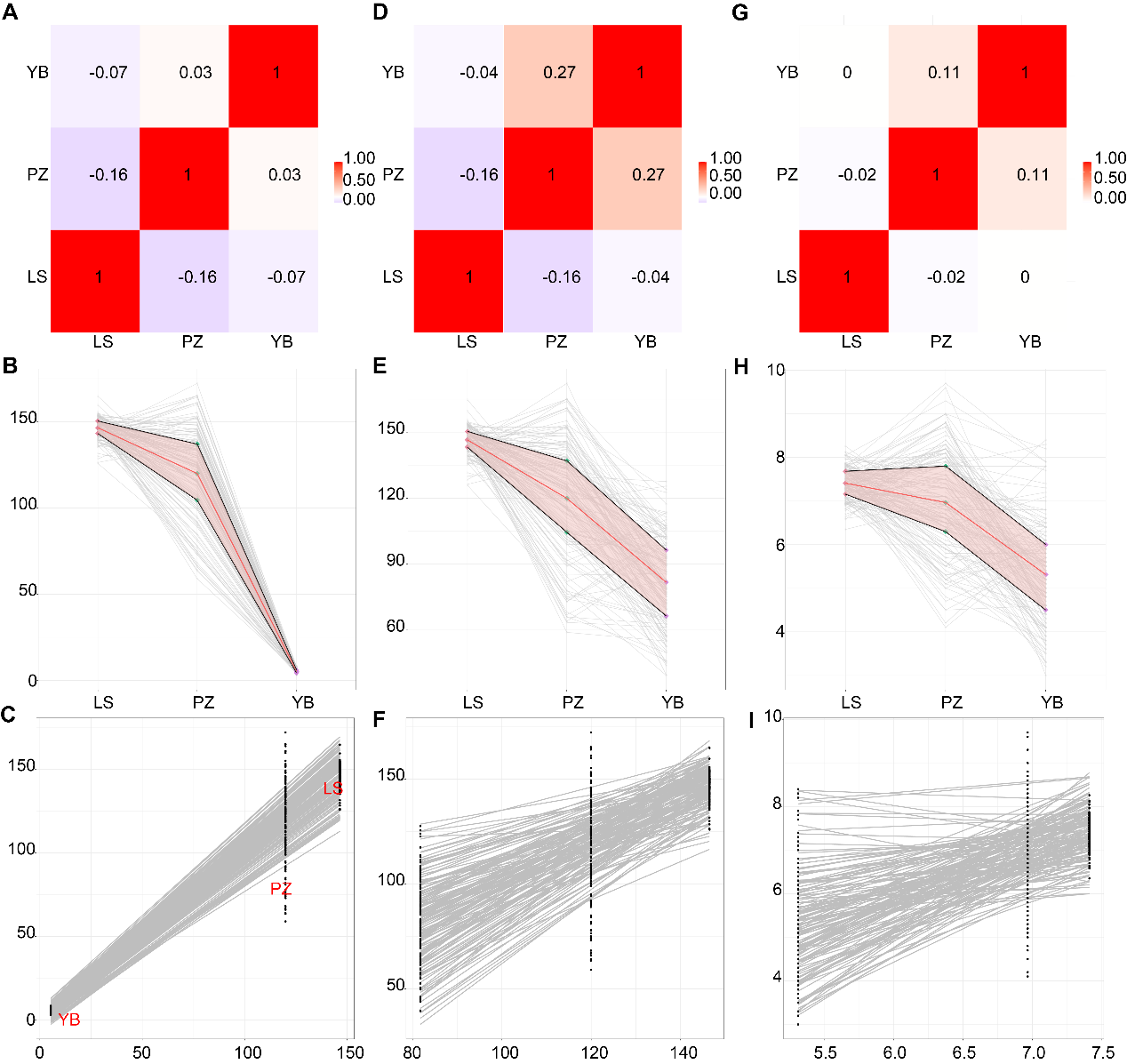


**Fig S8.** **Summary of three willow traits across three locations.** (A) Correlation of the defoliation time (DT) trait among locations. (B) Line chart of DT. The red line represents the mean DT, and the black lines represent the quartiles. The y-axis is in days. (C) Regression of DT on within site DT mean. The x-axis represents the mean DT for each location. (D) Correlation of plant height (PH) among locations. (E) Line chart of PH. The red line represents the mean PH, and the black lines represent the quartiles. The y-axis is in cm. (F) Regression of PH on within site PH mean. The x-axis represents the mean PH for each location. (G) Correlation of growth (G) among locations. (H) Line chart of G. The red line represents the mean G, and the black lines represent the quartiles. The y-axis is in cm. (I) Regression of G on within site G mean. The x-axis represents the mean G for each location.


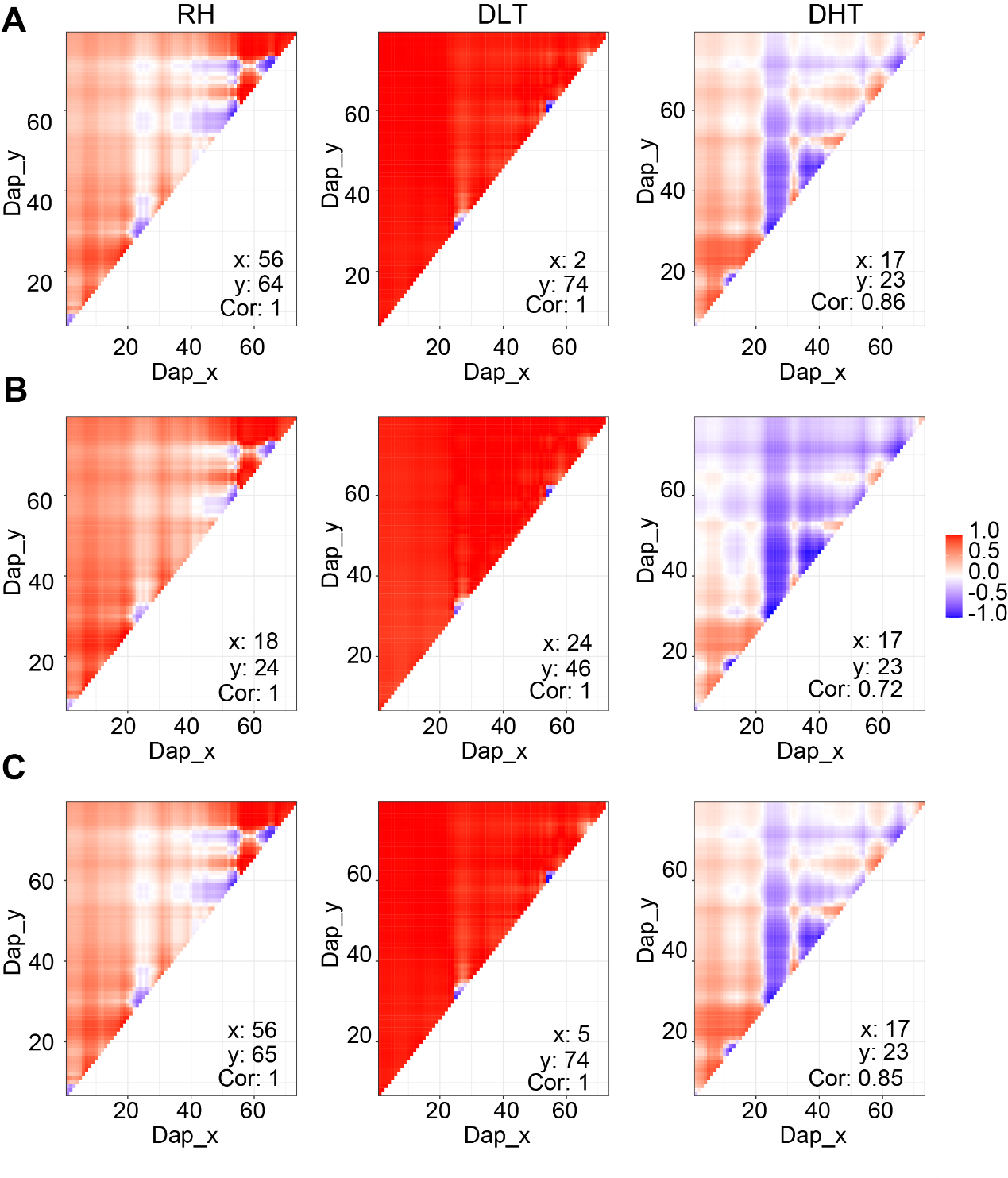


**Fig S9.** **Search for the environmental index by CERIS algorithm for the willow traits during the growth days with three environmental indexes.** (A) DTA; (B) PH; (C) G. The x-lab presents the search start day, while the y-lab presents the search end day. The x presents the strongest correlation to environmental means, which were selected as the start day, while the y presents the strongest correlation to environmental means, which were selected as the start day. The correlation of each window also has been presented, respectively. RH: relative humidity; DHT: daily highest temperature; DLT: daily lowest temperature.


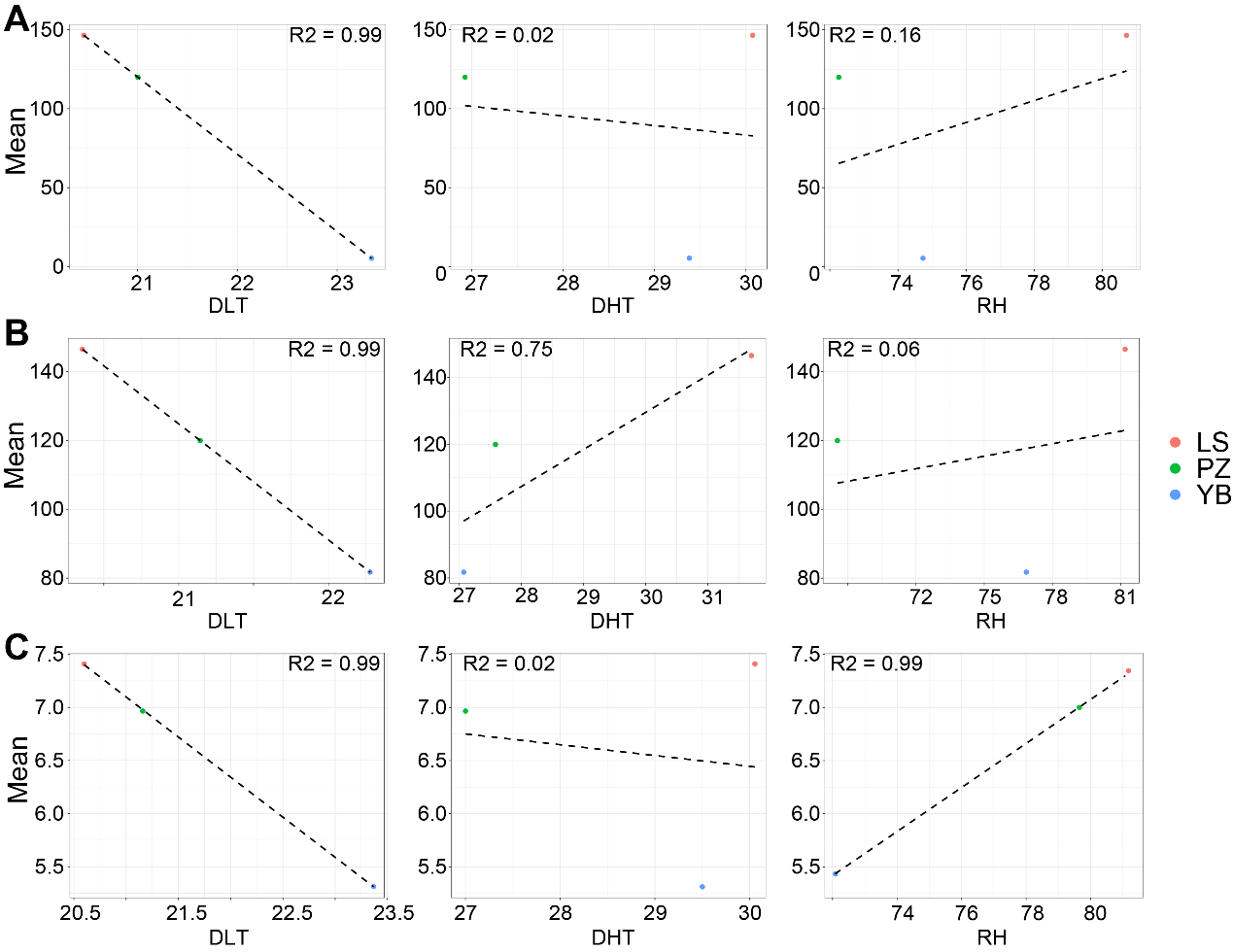


**Fig S10.** **Regression of willow traits on their identified environmental indexes.** For each trait, the x-axis shows the mean value of the environmental index (RH, DHT, or DLT) during its strongest correlated growth window. The y-axis shows the corresponding mean trait value for that same window. The R² value indicates the goodness of fit for each linear regression. (A) Defoliation time (DT). (B) Plant height (PH). (C) Growth (G).


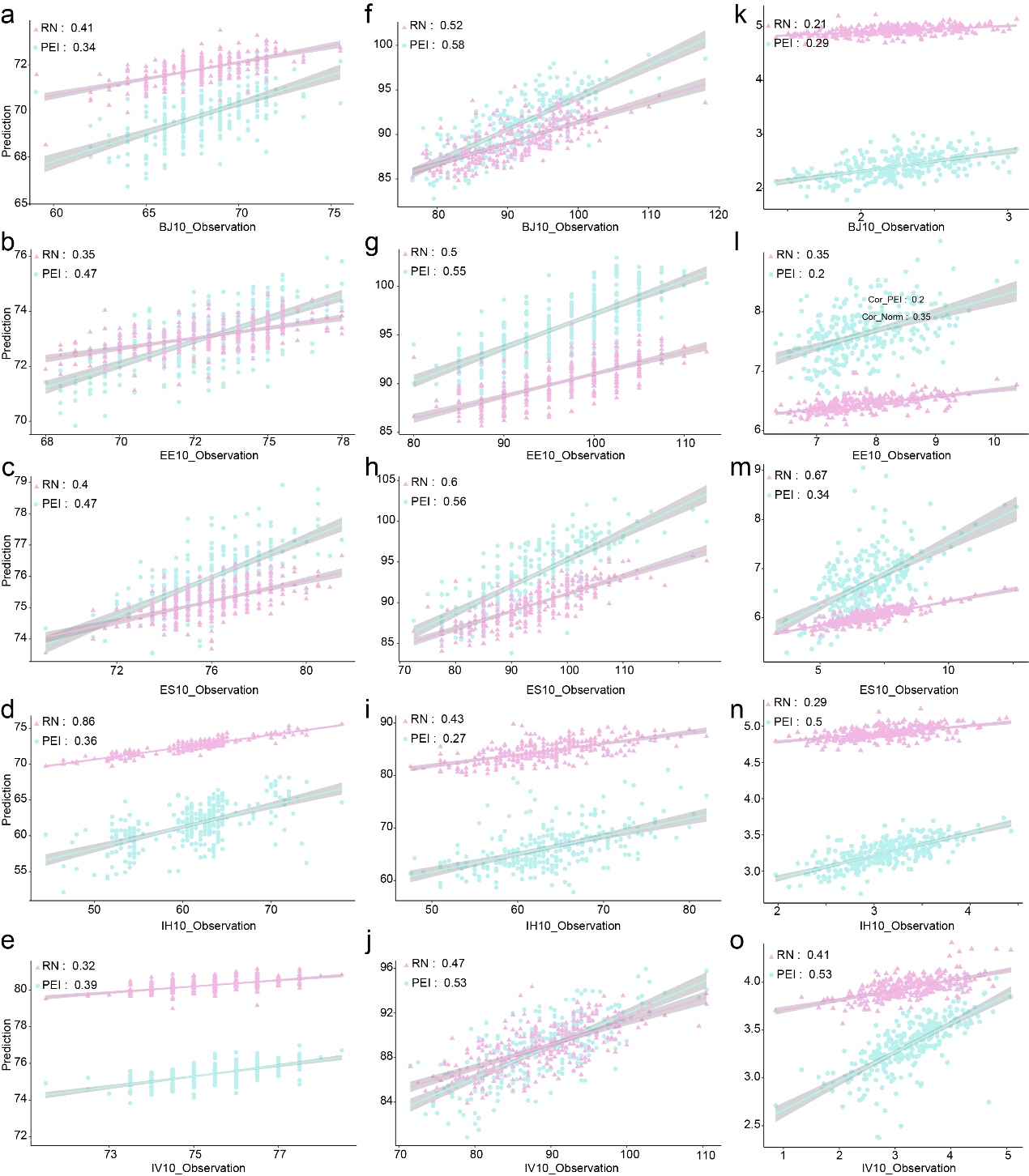


**Fig S11.** **Forecasting the performance of inbred wheat lines in Multi-Environment Trials (METs).** The prediction accuracy of three key trait-flowering time (FT; a-e), plant height (PH; f-j), and grain yield (YLD; k-o)-is evaluated across five different genomic or statistical prediction frameworks. Each framework is represented by a distinct color.


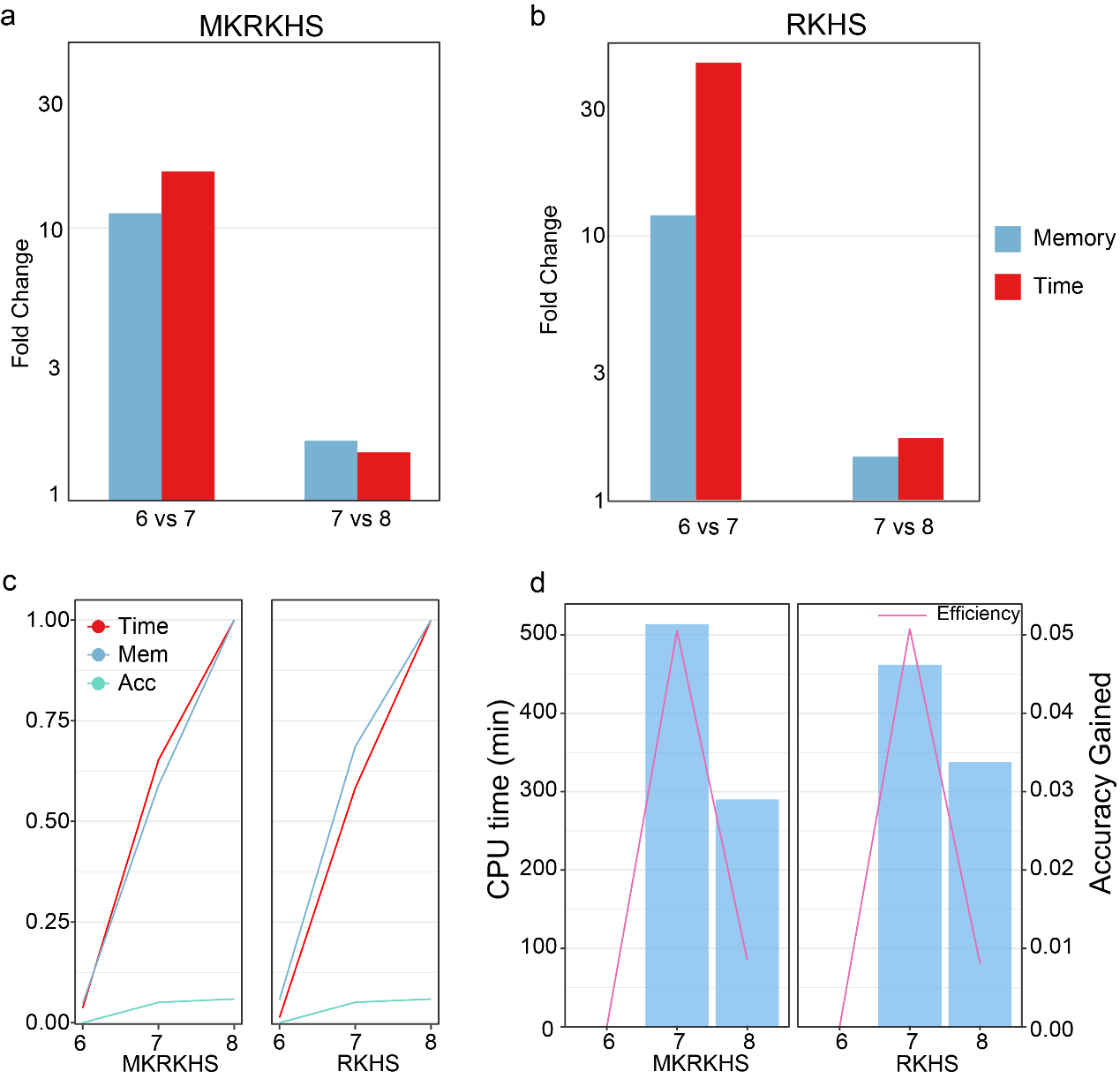


**Fig S12. Comparison of computational resources between semi-parametric methods across various dimensions.** (a-b) Computed time (min) and Memory (GB) used across various dimensions with MKRKHS and RKHS estimators; (c) Relative change (Time, Memory and Accuracy) across dimensions; (d) Curve of Diminishing Marginal Returns across dimensions.


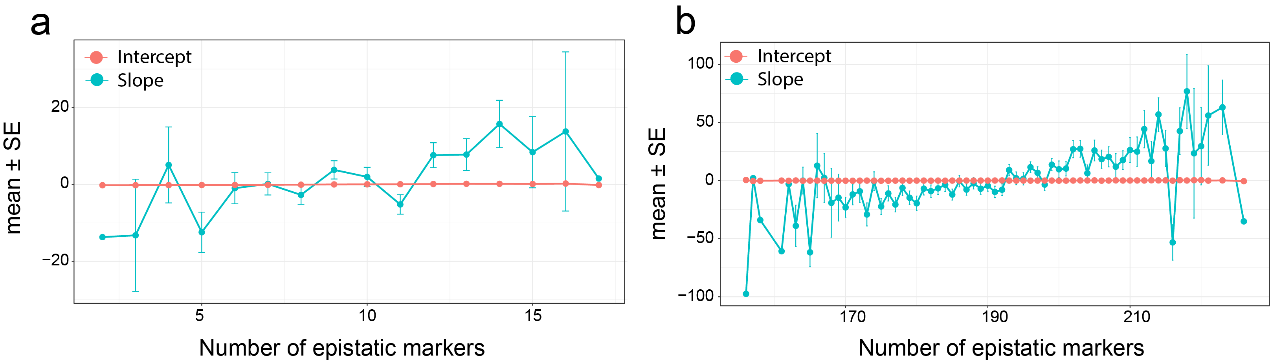


**Fig S13. Relationship between intercept and slope estimated from RN framework across individuals with varying numbers of epistatic markers (degree of epistatic interaction).** (a-b) Mean intercept and slope values along with standard error (SE) plotted against number of epistatic interactions in one environment and aggregated across 20 environments. Each dot represents a number of overlapping yeast strains with their intercept (red) and slope (green) estimated from RN framework plotted in y axis, and the number of epistatic interactions (degree of epistatic interaction) detected by Forsberg et al, Nature Genetics, 2017 and Zan et al, PLoS Genetics, 2020 in x axis.


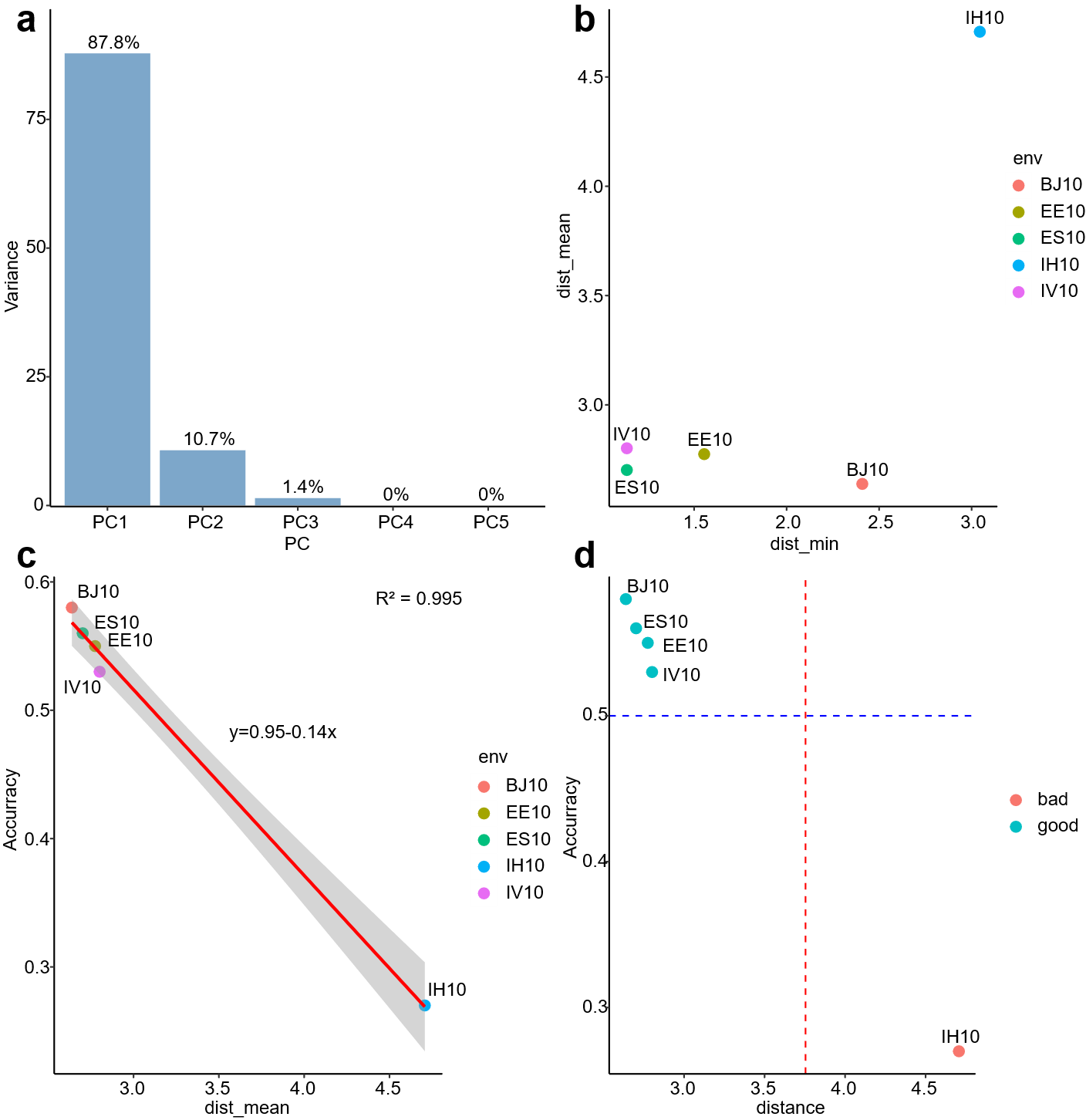


**Fig S14. A comparation between the RN and PEI frameworks in relation to environmental factor correlation and prediction stability.** (a) Variance explained by the first five principal components (PC1-PC5). (b) Scatter plot illustrates the relationship between minimum distance (Dist_min) and mean distance (Dist_mean) for five environmental conditions (BJ10, EE10, ES10, IH10, IV10). (c) Linear regression of accuracy versus mean distance (Dist_mean) across environments. (d) Classification of environmental conditions based on ROC analysis.


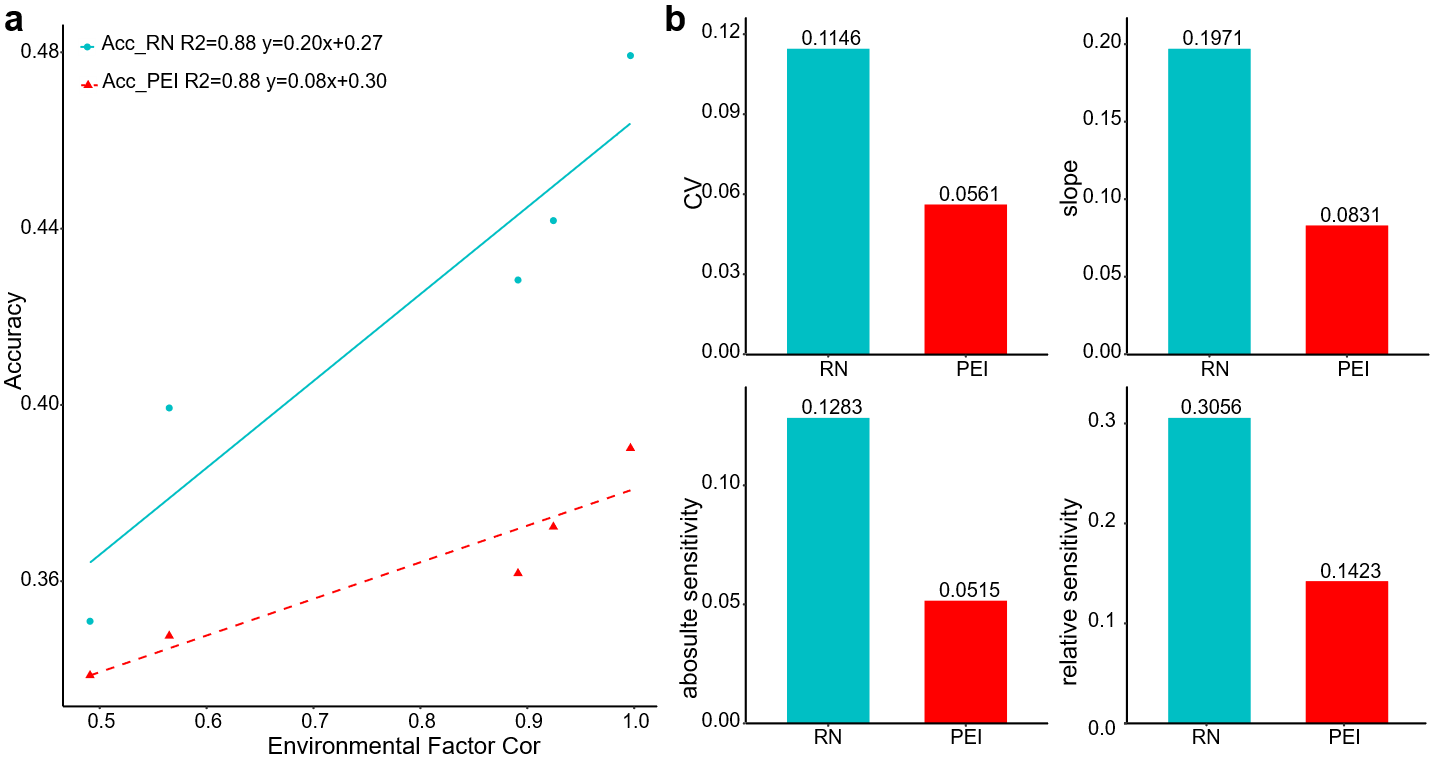


**Fig S15. Sensitivity analysis of the RN and PEI framework in relation to the selected environmental index.** (a) Relationship between predictive accuracy and the correlation of CERIS-derived environmental factor and the environmental gradient across environments. (b) Comparison of prediction stability metrics between the RN and PEI frameworks, including coefficient of variation (CV), slope of accuracy changes across environments, absolute sensitivity, and relative sensitivity.


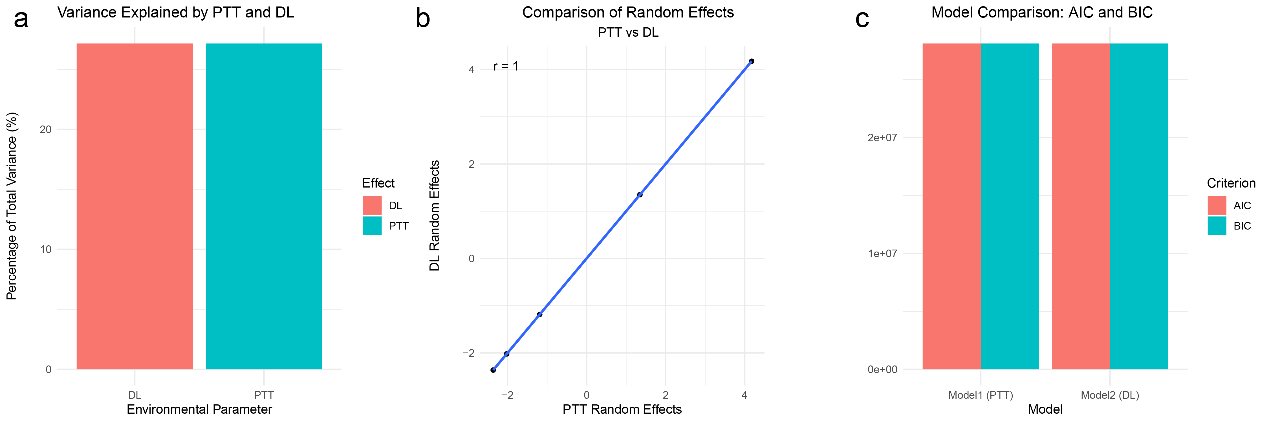


**Fig S16. Performances between models including a single environment factor (DL) and composed environment factors (PTT: photothermal time).** (a) Variance explained by two environmental factors. (b) Comparison of random effects between two environmental factors. (c) Model stability between two environmental factors. GDD from the date of sowing were calculated using the formula: GDD = (Tmax+ Tmin) /2−Tbase, where Tmax is the daily maximum temperature (86°F), Tmin is the daily minimum temperature, and Tbase is the base temperature for activity (50°F). If the Tmax exceeds 86°F, it is set to 86°F, and if the Tmin exceeds the Tbase, it is set to 50°F. PTT = GDD × DL.


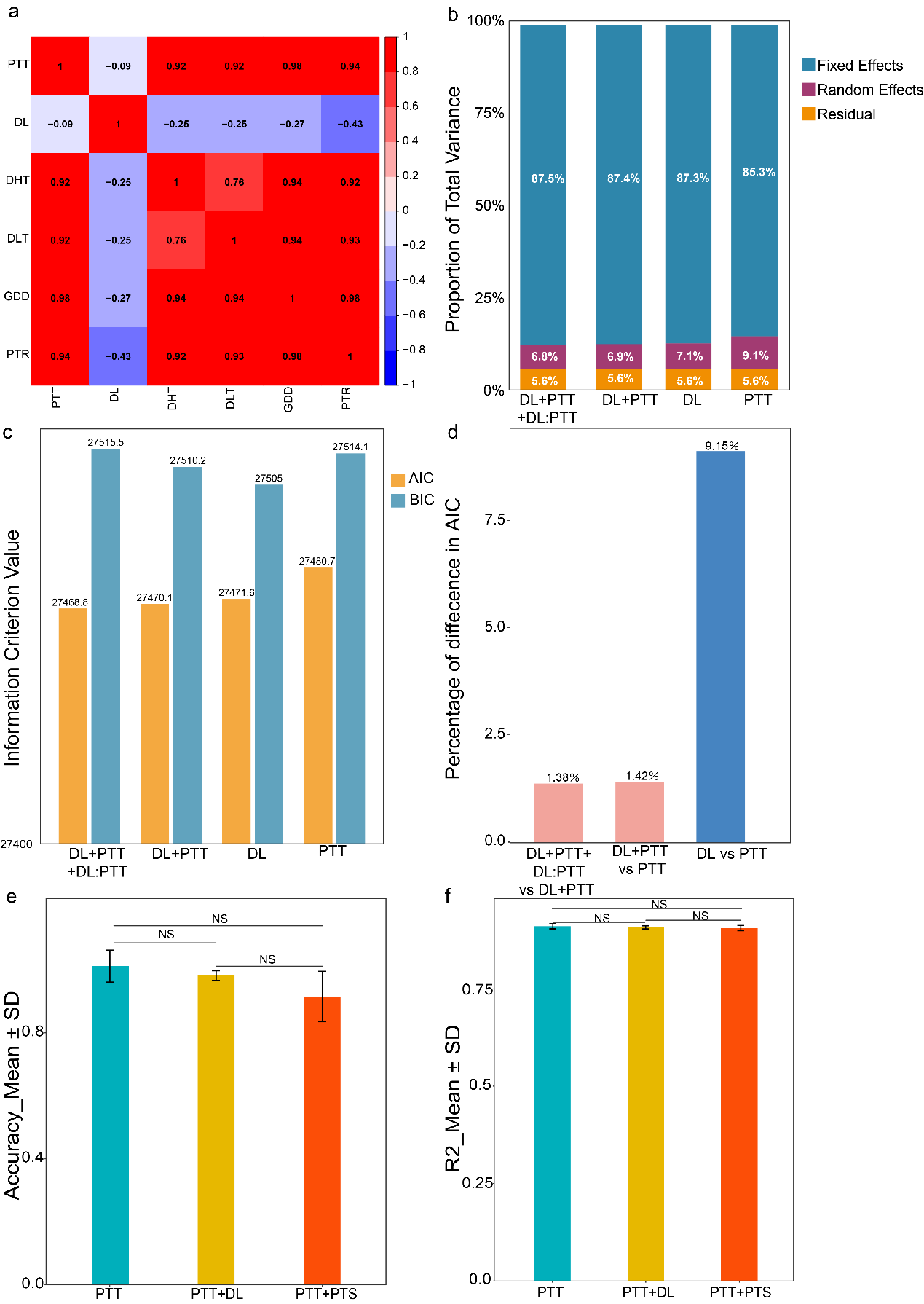


**Fig S17. A comparison between models with different number of environmental indexes.** (a) Collinearity between various environmental indexes; (b) Comparison of variance explained across different regression models with one or two most significant environmental indexes; (c) Comparison of AIC and BIC across different models; (d) Gain in AIC across different models. (e) Comparison of predicted accuracy across various environmental indexes based on PEI framework; (f) Comparison of model R2 across various models based on PEI framework.

**Note: DL: PTT presents the interaction between DL and PTT.**


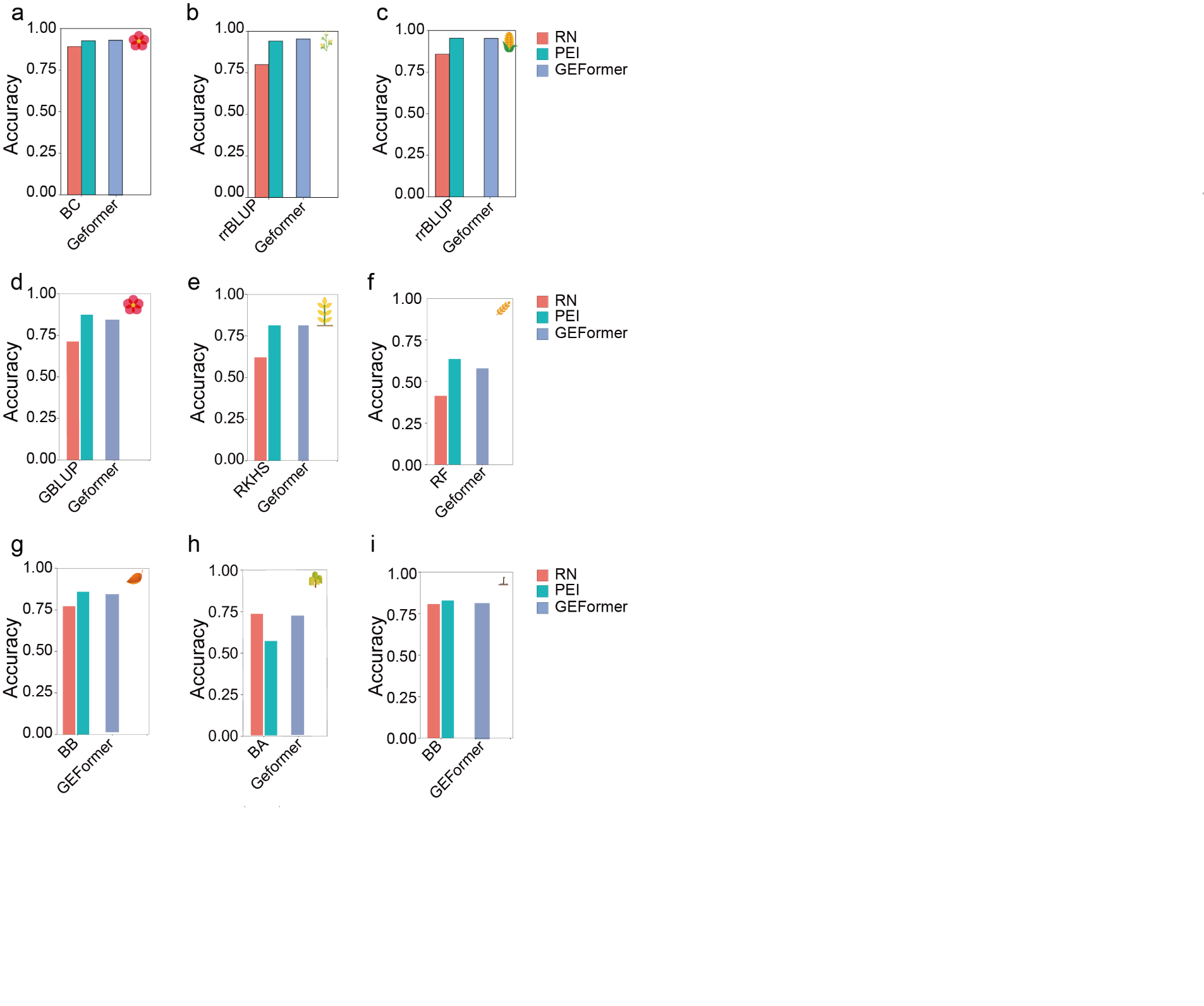


**Fig S18. Prediction accuracy for multiple traits in comparison to GEFormer.** (a-c) Performance of CUBIC maize population on Days to anthesis (DTA), Plant height (PH), and Ear weight (EW); (d-f) Performance of the CIMMYT inbred wheat population on Flowering time (FT), Plant height (PH), and Yield (YLD); (g-i) Performance of the F1 willow population on DT: Defoliation time; PH: Plant height; GD: Ground diameter.


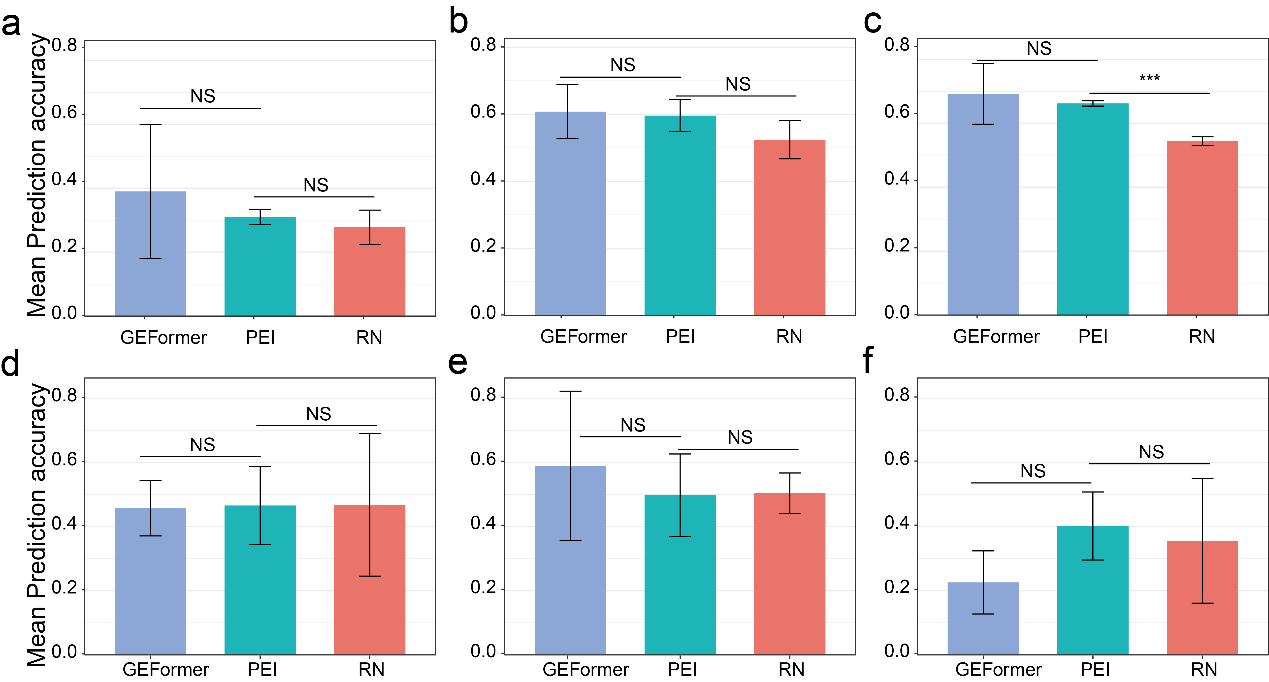


**Fig S19. Prediction accuracy for multiple traits in comparison to GEFormer in LOEO.** (a-c) Performance of CUBIC maize population on Ear weight (EW), Days to anthesis (DTA), and Plant height (PH); (d-f) Performance of the CIMMYT inbred wheat population on Flowering time (FT), Plant height (PH), and Yield (YLD).


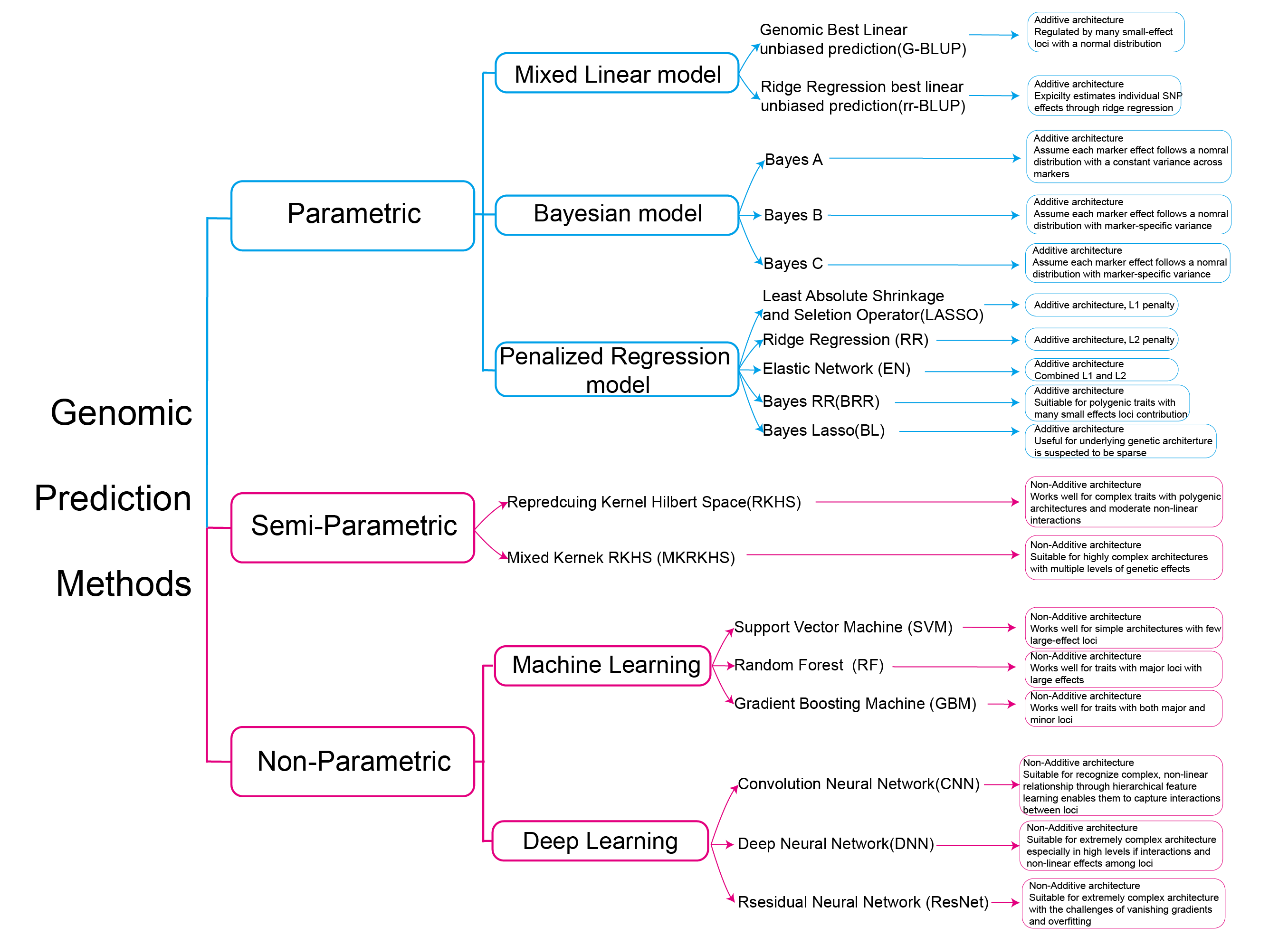


**Fig S20. An overview of various estimators.** Parametric estimators are additive, while others are non-additive.

**Supplementary Tables**

**Table S1. An overview of the CUBIC maize population locations.**

| ID | Site | Longitude | Latitude | Planting Date | Trial Year | Location |
| --- | --- | --- | --- | --- | --- | --- |
| 1 | BJ | 116.35 | 40.17 | 2014/5/13 | 2014 | Peking |
| 2 | HN | 114.02 | 35.45 | 2014/6/10 | 2014 | Henan |
| 3 | HB | 115.85 | 38.65 | 2014/6/10 | 2014 | Hebei |
| 4 | JL | 125.30 | 43.70 | 2014/5/7 | 2014 | Jining |
| 5 | LN | 123.55 | 42.05 | 2014/5/9 | 2014 | Liaoning |

**Table S2. An overview of the prediction accuracy in CUBIC maize population locations within three Frameworks of Days to anthesis.**

| Estimators | Framework | Mean Accuracy | Framework | Mean Accuracy | Framework | Mean Accuracy |
| --- | --- | --- | --- | --- | --- | --- |
| SVM | RN | 7.63E-01 | PEI | 7.98E-01 |  |  |
| rrBLUP | RN | 6.77E-01 | PEI | 8.50E-01 |  |  |
| RR | RN | 6.49E-01 | PEI | 7.61E-01 |  |  |
| RKHS | RN | 7.30E-01 | PEI | 8.25E-01 |  |  |
| RF | RN | 7.07E-01 | PEI | 7.93E-01 |  |  |
| MKRKHS | RN | 6.96E-01 | PEI | 6.86E-01 |  |  |
| LASSO | RN | 7.03E-01 | PEI | 8.63E-01 |  |  |
| GBM | RN | 6.97E-01 | PEI | 8.37E-01 | GEFormer | 8.33E-01 |
| GBLUP | RN | 6.59E-01 | PEI | 7.25E-01 |  |  |
| EN | RN | 8.18E-01 | PEI | 8.27E-01 |  |  |
| BRR | RN | 7.37E-01 | PEI | 7.10E-01 |  |  |
| BL | RN | 7.68E-01 | PEI | 7.86E-01 |  |  |
| BC | RN | 6.62E-01 | PEI | 8.06E-01 |  |  |
| BB | RN | 6.81E-01 | PEI | 7.43E-01 |  |  |
| BA | RN | 7.44E-01 | PEI | 7.44E-01 |  |  |

**Table S3. An overview of the prediction accuracy in CUBIC maize population locations within three Frameworks of Plant height.**

| Estimators | Framework | Mean Accuracy | Framework | Mean Accuracy | Framework | Mean Accuracy |
| --- | --- | --- | --- | --- | --- | --- |
| SVM | RN | 6.24E-01 | PEI | 6.30E-01 |  |  |
| rrBLUP | RN | 6.06E-01 | PEI | 6.28E-01 |  |  |
| RR | RN | 6.01E-01 | PEI | 6.02E-01 |  |  |
| RKHS | RN | 5.86E-01 | PEI | 5.96E-01 |  |  |
| RF | RN | 5.67E-01 | PEI | 5.96E-01 |  |  |
| MKRKHS | RN | 5.59E-01 | PEI | 5.77E-01 |  |  |
| LASSO | RN | 5.24E-01 | PEI | 5.72E-01 |  |  |
| GBM | RN | 5.23E-01 | PEI | 5.57E-01 | GEFormer | 6.32E-01 |
| GBLUP | RN | 4.73E-01 | PEI | 5.49E-01 |  |  |
| EN | RN | 4.47E-01 | PEI | 5.43E-01 |  |  |
| BRR | RN | 4.29E-01 | PEI | 5.33E-01 |  |  |
| BL | RN | 4.26E-01 | PEI | 5.19E-01 |  |  |
| BC | RN | 4.26E-01 | PEI | 5.17E-01 |  |  |
| BB | RN | 4.07E-01 | PEI | 4.64E-01 |  |  |
| BA | RN | 4.07E-01 | PEI | 3.81E-01 |  |  |

**Table S4. An overview of the prediction accuracy in CUBIC maize population locations within three Frameworks of Ear weight.**

| Estimators | Framework | Mean Accuracy | Framework | Mean Accuracy | Framework | Mean Accuracy |
| --- | --- | --- | --- | --- | --- | --- |
| SVM | RN | 4.09E-01 | PEI | 4.19E-01 |  |  |
| rrBLUP | RN | 5.15E-01 | PEI | 5.73E-01 |  |  |
| RR | RN | 4.42E-01 | PEI | 4.45E-01 |  |  |
| RKHS | RN | 4.16E-01 | PEI | 4.35E-01 |  |  |
| RF | RN | 5.26E-01 | PEI | 5.23E-01 |  |  |
| MKRKHS | RN | 4.54E-01 | PEI | 4.91E-01 |  |  |
| LASSO | RN | 3.85E-01 | PEI | 5.03E-01 |  |  |
| GBM | RN | 4.09E-01 | PEI | 5.50E-01 | GEFormer | 5.88E-01 |
| GBLUP | RN | 5.17E-01 | PEI | 5.48E-01 |  |  |
| EN | RN | 4.42E-01 | PEI | 5.33E-01 |  |  |
| BRR | RN | 4.25E-01 | PEI | 5.46E-01 |  |  |
| BL | RN | 3.86E-01 | PEI | 5.54E-01 |  |  |
| BC | RN | 4.12E-01 | PEI | 5.51E-01 |  |  |
| BB | RN | 3.59E-01 | PEI | 5.54E-01 |  |  |
| BA | RN | 3.62E-01 | PEI | 5.63E-01 |  |  |

**Table S5. An overview of the CIMMYT Inbred wheat population locations.**

| ID | Site | Longitude | Latitude | Planting Date | Trial Year | Location |
| --- | --- | --- | --- | --- | --- | --- |
| 1 | BJ10 | 23.46 | 90.23 | 12/10/2009 | 2009 | Joydebpur |
| 3 | EE10 | 25.5 | 32.6 | 12/22/2009 | 2009 | El Mat |
| 4 | ES10 | 27.17 | 31.32 | 12/20/2009 | 2009 | Sohag |
| 7 | IH10 | 15.26 | 75.07 | 12/7/2009 | 2009 | Dharwad |
| 9 | II10 | 22.37 | 75.5 | 12/10/2009 | 2009 | Indore |
| 11 | IK10 | 29.43 | 75.57 | 11/25/2009 | 2009 | Karnal |
| 13 | IL10 | 30.54 | 75.48 | 11/27/2009 | 2009 | Ludhiana |
| 15 | IV10 | 25.26 | 82.98 | 11/22/2009 | 2009 | Varanasi |
| 18 | MDF10 | 27.24 | -109.56 | 11/30/2009 | 2009 | Obr_DRT |

**Table S6. An overview of the prediction accuracy in CIMMYT Inbred wheat population locations within three Frameworks (Flowering Time).**

| Estimators | Framework | Mean Accuracy | Framework | Mean Accuracy | Framework | Mean Accuracy |
| --- | --- | --- | --- | --- | --- | --- |
| BA | RN | 7.75E-01 | PEI | 8.11E-01 |  |  |
| BB | RN | 6.88E-01 | PEI | 8.64E-01 |  |  |
| BC | RN | 6.59E-01 | PEI | 7.73E-01 |  |  |
| BL | RN | 7.42E-01 | PEI | 8.39E-01 |  |  |
| BRR | RN | 7.19E-01 | PEI | 8.06E-01 |  |  |
| EN | RN | 7.07E-01 | PEI | 6.97E-01 |  |  |
| GBLUP | RN | 7.15E-01 | PEI | 8.78E-01 |  |  |
| GBM | RN | 7.09E-01 | PEI | 8.51E-01 | GEFormer | 8.47E-01 |
| LASSO | RN | 6.70E-01 | PEI | 7.37E-01 |  |  |
| MKRKHS | RN | 8.32E-01 | PEI | 8.41E-01 |  |  |
| RF | RN | 7.49E-01 | PEI | 7.22E-01 |  |  |
| RKHS | RN | 7.80E-01 | PEI | 7.99E-01 |  |  |
| RR | RN | 6.73E-01 | PEI | 8.19E-01 |  |  |
| rrBLUP | RN | 6.92E-01 | PEI | 7.55E-01 |  |  |
| SVM | RN | 7.56E-01 | PEI | 7.56E-01 |  |  |

**Table S7. An overview of the prediction accuracy in CIMMYT Inbred wheat population locations within three Frameworks (Plant height).**

| Estimators | Framework | Mean Accuracy | Framework | Mean Accuracy | Framework | Mean Accuracy |
| --- | --- | --- | --- | --- | --- | --- |
| SVM | RN | 6.61E-01 | PEI | 7.32E-01 |  |  |
| rrBLUP | RN | 7.21E-01 | PEI | 6.96E-01 |  |  |
| RR | RN | 5.39E-01 | PEI | 7.01E-01 |  |  |
| RKHS | RN | 6.25E-01 | PEI | 8.17E-01 |  |  |
| RF | RN | 7.49E-01 | PEI | 8.07E-01 |  |  |
| MKRKHS | RN | 6.32E-01 | PEI | 8.15E-01 |  |  |
| LASSO | RN | 6.15E-01 | PEI | 7.06E-01 |  |  |
| GBM | RN | 6.31E-01 | PEI | 6.85E-01 | GEFormer | 8.17E-01 |
| GBLUP | RN | 7.08E-01 | PEI | 7.71E-01 |  |  |
| EN | RN | 6.19E-01 | PEI | 6.07E-01 |  |  |
| BRR | RN | 6.45E-01 | PEI | 8.14E-01 |  |  |
| BL | RN | 7.21E-01 | PEI | 8.00E-01 |  |  |
| BC | RN | 6.57E-01 | PEI | 6.95E-01 |  |  |
| BB | RN | 6.61E-01 | PEI | 7.62E-01 |  |  |
| BA | RN | 6.51E-01 | PEI | 7.90E-01 |  |  |

**Table S8. An overview of the prediction accuracy in CIMMYT Inbred wheat population locations within three Frameworks (Yield).**

| Estimators | Framework | Mean Accuracy | Framework | Mean Accuracy | Framework | Mean Accuracy |
| --- | --- | --- | --- | --- | --- | --- |
| BA | RN | 2.50E-01 | PEI | 3.02E-01 |  |  |
| BB | RN | 3.70E-01 | PEI | 3.58E-01 |  |  |
| BC | RN | 3.35E-01 | PEI | 3.94E-01 |  |  |
| BL | RN | 3.14E-01 | PEI | 3.75E-01 |  |  |
| BRR | RN | 3.08E-01 | PEI | 4.36E-01 |  |  |
| EN | RN | 3.99E-01 | PEI | 5.11E-01 |  |  |
| GBLUP | RN | 3.67E-01 | PEI | 2.25E-01 |  |  |
| GBM | RN | 3.84E-01 | PEI | 5.16E-01 | GEFormer | 5.81E-01 |
| LASSO | RN | 2.19E-01 | PEI | 3.75E-01 |  |  |
| MKRKHS | RN | 5.69E-01 | PEI | 3.59E-01 |  |  |
| RF | RN | 4.15E-01 | PEI | 6.38E-01 |  |  |
| RKHS | RN | 2.42E-01 | PEI | 3.09E-01 |  |  |
| RR | RN | 3.26E-01 | PEI | 2.65E-01 |  |  |
| rrBLUP | RN | 2.41E-01 | PEI | 2.05E-01 |  |  |
| SVM | RN | 2.24E-01 | PEI | 4.24E-01 |  |  |

**Table S9. An overview of the F1 willow population location**

| ID | Site | Longitude | Latitude | Planting Date | Trial Year | Location |
| --- | --- | --- | --- | --- | --- | --- |
| 1 | LS | 103.35 | 29.62 | 2022/5/13 | 2022 | Leshan |
| 2 | PZ | 104.00 | 31.17 | 2022/5/13 | 2022 | Pengzhou |
| 3 | YB | 104.61 | 28.79 | 2022/5/13 | 2022 | Yibin |

**Table S10. An overview of the prediction accuracy in F1 willow population locations within three Frameworks (Defoliation time).**

| Estimators | Framework | Mean Accuracy | Framework | Mean Accuracy | Framework | Mean Accuracy |
| --- | --- | --- | --- | --- | --- | --- |
| BA | RN | 6.13E-01 | PEI | 6.29E-01 |  |  |
| BB | RN | 7.73E-01 | PEI | 8.60E-01 |  |  |
| BC | RN | 6.62E-01 | PEI | 6.68E-01 |  |  |
| BL | RN | 6.25E-01 | PEI | 6.53E-01 |  |  |
| BRR | RN | 7.89E-01 | PEI | 7.84E-01 |  |  |
| EN | RN | 6.81E-01 | PEI | 7.36E-01 |  |  |
| GBLUP | RN | 5.78E-01 | PEI | 7.55E-01 |  |  |
| GBM | RN | 6.14E-01 | PEI | 8.26E-01 | GEFormer | 8.30E-01 |
| LASSO | RN | 7.75E-01 | PEI | 8.22E-01 |  |  |
| MKRKHS | RN | 6.62E-01 | PEI | 7.99E-01 |  |  |
| RF | RN | 6.37E-01 | PEI | 8.19E-01 |  |  |
| RKHS | RN | 5.79E-01 | PEI | 8.31E-01 |  |  |
| RR | RN | 6.19E-01 | PEI | 8.26E-01 |  |  |
| rrBLUP | RN | 5.39E-01 | PEI | 8.30E-01 |  |  |
| SVM | RN | 5.43E-01 | PEI | 8.45E-01 |  |  |

**Table S11. An overview of the prediction accuracy in F1 willow population locations within three Frameworks (Plant height).**

| Estimators | Framework | Mean Accuracy | Framework | Mean Accuracy | Framework | Mean Accuracy |
| --- | --- | --- | --- | --- | --- | --- |
| SVM | RN | 5.88E-01 | PEI | 5.09E-01 |  |  |
| rrBLUP | RN | 6.01E-01 | PEI | 5.00E-01 |  |  |
| RR | RN | 5.65E-01 | PEI | 4.98E-01 |  |  |
| RKHS | RN | 7.13E-01 | PEI | 4.79E-01 |  |  |
| RF | RN | 7.17E-01 | PEI | 5.33E-01 |  |  |
| MKRKHS | RN | 6.92E-01 | PEI | 5.56E-01 |  |  |
| LASSO | RN | 5.06E-01 | PEI | 4.45E-01 |  |  |
| GBM | RN | 6.85E-01 | PEI | 5.47E-01 | GEFormer | 7.18E-01 |
| GBLUP | RN | 6.06E-01 | PEI | 5.64E-01 |  |  |
| EN | RN | 5.60E-01 | PEI | 5.27E-01 |  |  |
| BRR | RN | 6.64E-01 | PEI | 6.17E-01 |  |  |
| BL | RN | 6.23E-01 | PEI | 5.62E-01 |  |  |
| BC | RN | 7.03E-01 | PEI | 5.79E-01 |  |  |
| BB | RN | 6.79E-01 | PEI | 5.48E-01 |  |  |
| BA | RN | 7.31E-01 | PEI | 5.67E-01 |  |  |

**Table S12. An overview of the prediction accuracy in F1 willow population locations within three Frameworks (Ground diameter).**

| Estimators | Framework | Mean Accuracy | Framework | Mean Accuracy | Framework | Mean Accuracy |
| --- | --- | --- | --- | --- | --- | --- |
| BA | RN | 6.36E-01 | PEI | 6.55E-01 |  |  |
| BB | RN | 8.08E-01 | PEI | 8.29E-01 |  |  |
| BC | RN | 7.31E-01 | PEI | 6.47E-01 |  |  |
| BL | RN | 7.64E-01 | PEI | 7.80E-01 |  |  |
| BRR | RN | 7.43E-01 | PEI | 7.49E-01 |  |  |
| EN | RN | 7.20E-01 | PEI | 5.48E-01 |  |  |
| GBLUP | RN | 7.49E-01 | PEI | 8.03E-01 |  |  |
| GBM | RN | 7.78E-01 | PEI | 7.15E-01 | GEFormer | 8.19E-01 |
| LASSO | RN | 5.98E-01 | PEI | 5.74E-01 |  |  |
| MKRKHS | RN | 8.22E-01 | PEI | 6.80E-01 |  |  |
| RF | RN | 4.91E-01 | PEI | 6.67E-01 |  |  |
| RKHS | RN | 8.11E-01 | PEI | 6.20E-01 |  |  |
| RR | RN | 5.94E-01 | PEI | 7.43E-01 |  |  |
| rrBLUP | RN | 6.54E-01 | PEI | 7.64E-01 |  |  |
| SVM | RN | 6.00E-01 | PEI | 5.62E-01 |  |  |

**Table S13. An overview of computational time of GEFormer on CPU and GPU devices in CUBIC population.**

| Device | Total time (5 environments) | Hyper time (5 environments) |
| --- | --- | --- |
| CPU | 9d | 7d |
| GPU (24Gb, 1 core) | 17h | 12h |

Notes: the efficiency on GPU device influenced by GPU memory and core numbers

**Table S14. An overview hyperparameter of GEFormer on various populations.**

| Populations | Traits | Batch | Dropout | Depth | Neurons1 | Neurons2 | Learning rate |
| --- | --- | --- | --- | --- | --- | --- | --- |
|  | DTA | 245 | 2.99E-01 | 13 | 862 | 193 | 2.92E-03 |
| CUBIC | PH | 111 | 3.20E-01 | 15 | 565 | 102 | 1.83E-05 |
|  | EW | 154 | 4.22E-01 | 1 | 634 | 246 | 1.16E-04 |
|  | FT | 70 | 2.04E-01 | 3 | 870 | 99 | 1.67E-04 |
| CIMMIY | PH | 158 | 3.12E-01 | 5 | 708 | 162 | 1.90E-04 |
|  | YLD | 244 | 2.89E-01 | 14 | 578 | 173 | 2.91E-07 |
|  | GD | 42 | 4.41E-01 | 2 | 712 | 81 | 2.75E-05 |
| F1 Willow | PH | 129 | 4.82E-01 | 14 | 637 | 63 | 2.48E-07 |
|  | DT | 56 | 2.72E-01 | 11 | 989 | 228 | 9.58E-03 |

Notes: Searched by Optuna
