# Supplementary material for: MMGS: a novel genomic prediction framework to integrate genotype, environment and their interactions for multi-environment breeding trials
Source: Hortic Res. 2026 Mar 16;13(5):uhag035. doi: 10.1093/hr/uhag035 (PMC13150855; doi:10.1093/hr/uhag035)
Supplement: Web_Material_uhag035 [file web_material_uhag035.zip › Supplementary_Notes-20251218zyj.docx]

**Supplementary Notes**

**Comparsion of the ability to capture the epistatic effect between PEI and RN**

To evaluate whether the Reaction Norm (RN) framework is able to capture epistatic signals without explicitly modeling gene-gene interactions, we analyzed the *Saccharomyces cerevisiae* genotype-phenotype dataset published in *Nature Genetics* (Noble and Grigoriev) ^1^. Genotype and multi-environment phenotype data were obtained from the NG yeast dataset. Single-nucleotide polymorphisms (SNPs) were first standardized and subjected to standard quality control procedures, including filtering based on missingness and minor allele frequency. Phenotypic records across environments were subsequently pre-processed using linear mixed models or normalization approaches to derive individual-specific reaction norm parameters, namely intercepts and slopes. Environmental covariates were represented by the mean phenotypic values of each environment.

To assess the relationship between reaction norm parameters and epistatic architecture, each individual was characterized by the number of markers previously reported to exhibit epistatic effects. Scatter plots were generated to visualize the associations between reaction norm intercepts or slopes and the degree of epistatic interaction for each individual. Linear regression analyses were then performed to quantify the strength and direction of these relationships.

This analysis allows us to examine whether variation in reaction norm parameters reflects underlying epistatic genetic contributions, thereby providing empirical evidence that the RN framework can capture epistasis-related signals indirectly, even in the absence of explicit gene-gene interaction terms.

**Benchmarking of the GEFormer method**

To provide a representative comparison with state-of-the-art approaches for multi-environment genomic prediction, we benchmark against a recently published framework, GEFormer ^2^. GEFormer adopts an MLP-based architecture that integrates genomic datasets and environmental indices within a unified representation-learning framework. Briefly, genetic markers and environmental covariates are first embedded into latent feature spaces, followed by multiple stacked attention layers that enable the model to capture high-order, non-linear interactions between genomic loci and multiple environmental factors. Prediction is obtained through fully connected layers that map the learned joint representations to phenotypic values.

We followed the original design of GEFormer and applied it to our datasets. Hyperparameter selection was performed using the Optuna Bayesian optimization library, with a search conducted on the training set for various traits of three populations to obtain optimal configuration. All GEFormer analyses were implemented in Python3.9.19 using PyTorch. A complete description of training hyperparameters across various datasets in the supplementary tables (Table S14). All

benchmarking analyses were performed under identical cross-validation schemes and using the same training-testing partitions. This ensures that observed performance differences primarily reflect model assumptions and representational capacity, rather than advantages arising from unequal tuning effort.

**Evaluating when prediction will be unstable in leave one environment out prediction**

For each target environment, we computed (i) the minimum distance to any other environment $d_{min}$ ; (ii) the mean distance to all other environments $d_{mean}$. These metrics were used as quantitative indicators of environmental continuity.

$d(e,u)=||S_{e} - S_{u} ||_{2}, e,u \in\{1,\ldots,m\}$（1）

Environments were assigned category labels based on a threshold, and sensitivity and specificity were calculated across a range of possible distance values. ROC curves were generated by evaluating the true positive rate and false positive rate at each distance threshold. The optimal distance threshold was determined using the Youden index ^3^.

$Youden index=\max_{\tau}(TPR(\tau)-FPR(\tau))$ (2)

Where $\tau$ denotes distance thresholds. This analysis allowed us to determine an approximate environmental distance beyond which performance becomes unreliable.

**Reference**

1 Forsberg SKG, Bloom JS, Sadhu MJ, Kruglyak L, Carlborg Ö. Accounting for genetic interactions improves modeling of individual quantitative trait phenotypes in yeast. *Nat Genet* 2017; **49**: 497–503.

2 Yao Z *et al.* GEFormer: a Genomic Prediction Method of Genotype-Environment Interaction in Maize by Integrating Gating Mechanism MLP and Linear Attention Mechanism. *Molecular Plant* 2025.

3 Aayushi Tandon, Amit Awasthi, Kanhu Charan Pattnayak & Sumanta Das. (2025) Rainfall Variability and Rising Extremes in Urbanizing Himalayan Foothills: A Machine Learning and data-driven Exploration of Hydroclimatic Shifts in Uttarakhand, India. Earth Systems and Environment.
